# Spinal Glycine Receptor Alpha 3 Cells Communicate Sensations of Chemical Itch in Hairy Skin

**DOI:** 10.1523/JNEUROSCI.1585-23.2024

**Published:** 2024-03-29

**Authors:** Hannah M. Weman, Mikaela M. Ceder, Aikeremu Ahemaiti, Kajsa A. Magnusson, Katharina Henriksson, Linn Andréasson, Malin C. Lagerström

**Affiliations:** Department of Immunology, Genetics and Pathology, Uppsala University, Uppsala 75108, Sweden

**Keywords:** chemogenetics, glycine, itch, retrograde tracing, spinal cord

## Abstract

Glycinergic neurons regulate nociceptive and pruriceptive signaling in the spinal cord, but the identity and role of the glycine-regulated neurons are not fully known. Herein, we have characterized spinal glycine receptor alpha 3 (*Glra3*) subunit-expressing neurons in *Glra3*-Cre female and male mice. *Glra3*-Cre(+) neurons express *Glra3*, are located mainly in laminae III–VI, and respond to glycine. Chemogenetic activation of spinal *Glra3*-Cre(+) neurons induced biting/licking, stomping, and guarding behaviors, indicative of both a nociceptive and pruriceptive role for this population. Chemogenetic inhibition did not affect mechanical or thermal responses but reduced behaviors evoked by compound 48/80 and chloroquine, revealing a pruriceptive role for these neurons. Spinal cells activated by compound 48/80 or chloroquine express *Glra3*, further supporting the phenotype. Retrograde tracing revealed that spinal *Glra3*-Cre(+) neurons receive input from afferents associated with pain and itch, and dorsal root stimulation validated the monosynaptic input. In conclusion, these results show that spinal *Glra3*(+) neurons contribute to acute communication of compound 48/80- and chloroquine-induced itch in hairy skin.

## Significance Statement

Spinal glycinergic neurons regulate itch (pruriception), suggesting that components of the glycinergic system have great potential as drug targets to treat pruritus. Nonetheless, thus far, the pruriceptive roles of any of the glycine receptor (GLR) subunits have not been evaluated. Here, we successfully linked the *Glra3*-Cre populations to a pro-pruriceptive role in itch, indicating that GLRA3-expressing neurons may be a potential novel target for itch treatment. The spontaneous stomping and guarding behaviors observed from activating the *Glra3*-Cre populations are indicative of a role in sensory hypersensitivity and hence, raises questions regarding the hypersensitivity involvement of these populations for future investigations.

## Introduction

Spinal somatosensory circuits transmitting the sensation of pain and itch from the body are regulated locally by inhibitory inputs, including glycinergic transmission (Beyer et al., 1985; Yamamoto and Yaksh, 1993; Takazawa et al., 2017; Freitag et al., 2019). For instance, ablation of glycine transporter 2 (GLYT2) neurons results in mechanical, heat, and cold hyperalgesia and behaviors associated with persistent itch, for example, extensive localized biting (Foster et al., 2015). Conversely, selective activation of GLYT2 neurons in vivo reduces the sensitivity to mechanical-, heat-, and cold-induced pain and the behavioral responses against chloroquine and histamine, suggesting that the glycinergic system is essential for controlling pain and itch transmission (Foster et al., 2015). In addition, the glycinergic system is activated by nociceptive counter stimuli, which decrease itch transmission in the spinal cord (Akiyama et al., 2011).

Glycinergic receptors (GLRs) are ligand-gated ion channels, which induce inward hyperpolarizing chloride currents upon binding of glycine (Lynch, 2004; Zeilhofer, 2005; Lein et al., 2007; Dutertre et al., 2012). In mice, the glycine receptor alpha 3 (*Glra3*) gene is expressed in both excitatory and inhibitory spinal dorsal horn neuronal clusters (Häring et al., 2018; Zeisel et al., 2018; Ceder et al., 2023) and GLRA3 immunoreactivity is detected in the dorsal (Harvey et al., 2004; Wang et al., 2018; Werynska et al., 2021) and ventral horns of the spinal cord (Harvey et al., 2004; Wang et al., 2018). The other *Glr* genes, *Glra1*, *Glra2*, *Glra4*, and *Glrb*, are also expressed in the dorsal horn of the spinal cord (Groemer et al., 2022). In addition to the expression in the spinal cord, *Glra3* is detected in the amygdala, hypothalamus, nucleus accumbens, tegmentum, and brainstem, but not in the dorsal root ganglia (DRG; Lein et al., 2007; Usoskin et al., 2015; McCracken et al., 2017; Häring et al., 2018; Tudeau et al., 2020; San Martin et al., 2021; Groemer et al., 2022). Expression analyses have shown that spinal cord injury decreases levels of GLRA3 in the dorsal spinal cord, whereas zymosan A-induced inflammation increases GLRA3 levels (Berrocal et al., 2014; Mariqueo, 2020). Additionally, in an endometriosis mouse model, *Glra3* expression was found to be upregulated in the insula (Li et al., 2018), emphasizing this receptor subunit's role in pain and its potential as a novel pain treatment.

Thus far, studies have focused on examining the nociceptive role of the GLRA3 subunit. Herein, we investigated the molecular and electrophysiological characteristics, along with the sensory role of spinal *Glra3*-expressing cells in pruriceptive, mechanical, and thermal transmission, using a transgenic *Glra3*-Cre mouse line and *fos* measurements. Moreover, we established neuronal inputs to the population, using replication deficient rabies tracing and dorsal root stimulations.

## Materials and Methods

### Animals

Procedures related to the mice used in this study were approved by the local animal research ethical committee (Uppsala djurförsöksetiska nämnd) and followed the Swedish Animal Welfare Act [Svensk författningssamling (SFS) 2018:1192], the Swedish Animal Welfare Ordinance (SFS 2019:66), and the Regulations and General Advice for Laboratory Animals (SJVFS 2019:9, Saknr L 150), permit numbers: 5.8.18-01428/2023, 5.2.18-17971/2019, 5.8.18-11551/2019, 5.8.18-19421/2019, 5.8.18-01217/2019, 5.8.18-01503/2023 and 5.8.18-03266/2023. The constitutive knock-in *Glra3*-Cre mouse line was generated by Cyagen, with the homology arms having been amplified from a bacterial artificial chromosome (BAC), for which the *Glra3* gene is located on chromosome 8 (GenBank: NM_080438.2, Ensembl: ENSMUS00000038257). *Glra3*-Cre(+) mice were crossed with C57BL/6J mice (Taconic) and *tdTomato* reporter mice [Gt(ROSA)26Sor^tm14(CAG-tdTomato)Hze^, Allen Brain Institute]. The *Glra3*-Cre allele was kept hemizygous and both female and male mice were included in the analyses, unless otherwise stated. The mice were housed with littermates in ∼501 cm^2^ cages (maximum five mice per cage) in room temperature (RT) ranging between 20 and 24°C and humidity of 45–65% on a 12 h light/dark cycle with lights on at 6 A.M. All mice were provided food (Diet Pellets, Scanbur) and tap water *ad libitum*.

### Genotyping by polymerase chain reaction

Tissue biopsies from ear marking, taken at the age of 3–4 weeks, were incubated in 50 µl of buffer, consisting of 25 mM NaOH and 200 µM ethylenediaminetetra-acetic acid (EDTA), in a shaking block (BIOER Mixing Block MB-102, 300 speed) at 96°C for 25 min, whereafter the samples were neutralized with 50 µl of Tris-HCl (40 mM), pH 8.0. The following primers were used to identify *Cre* and *tdTomato*, respectively; *Cre* 5′-acgagtgatgaggttcgcaaga-3′ (forward, mutant allele), 5′-accgacgatgaagcatgtttag-3′ (reverse, mutant allele), *tdTomato* 5′-ctgttcctgtacggcatgg-3′ (forward, mutant allele), 5′-ggcattaaagcagcgtatcc-3′ (reverse, mutant allele), 5′-aagggagctgcagtggagta-3′ (forward, wild-type allele), 5′-ccgaaaatctgtgggaagtc-3′ (reverse, wild-type allele).

### Spinal cord viral injections

The viral injections into the spinal cord were performed for the chemogenetic sensory tests, monosynaptic retrograde tracing, and the electrophysiological recordings of adult *Glra3*-Cre(+) neurons. The injections were performed as previously described (Freitag et al., 2019); in brief, *Glra3*-Cre mice (>6 weeks old) were initially anesthetized in a 4% isoflurane (FORANE, Baxter) box. When fully anesthetized, the mice were moved to a stereotaxic frame with a breathing mask, where the isoflurane concentration was kept at 1.5–2% throughout the entire procedure. To prevent eye damage, Oftagel was applied (Santen Oy), and the body temperature was monitored and maintained at 35–37°C using a heating pad (FHC). Adjacent to the incision sites, the mice were administered subcutaneously with bupivacaine (Marcain, 2 mg/kg, AstraZeneca). For postsurgery analgesia, the mice were administered subcutaneously with carprofen (Norocarp vet, 5 mg/kg, N-vet, or Rimadyl Bovis vet, 4 mg/kg, Zoetis Finland Oy). Within 24 h postsurgery, the mice were again administered 4–5 mg/kg carprofen for postsurgery analgesia. The dorsal skin was shaved and cleaned with sterile saline (B Braun Medical) and chlorhexidine (Fresenius Kabi) before a 1 cm skin incision was made to expose the T13 and L1 vertebrae. Sterile saline was continuously applied to keep the tissue moist. The connective tissue was gently separated along these vertebrae, and a clamp was inserted ventral of the L1 transverse process for stabilization of the spine. When stabilized, the posterior longitudinal ligament and ligamentum flavum connecting T13 and L1 were cut to expose the spinal cord. Thereafter, 500 nl of the respective viral vector [*AAV8.hSyn-DIO-hM3D(Gq)-mCherry* (Krashes et al., 2011), *AAV8.hSyn-DIO-mCherry*, or *AAVDJ.EF1a-DIO-HTB*; please see Table 1 for detailed information] was injected into the L5/L6 spinal dorsal horn (as caudal as possible from zeroed midline, ML, 0.4 mm; DV, 0.4 mm; with needle eye directed rostrally), using a 10 µl NanoFil Hamilton syringe (World Precision Instruments) with a 34 g beveled needle (World Precision Instruments), monitored by a micro syringe pump controller (World Precision Instruments) at 50 nl/min. For injections of *AAV8.hSyn-DIO-hM4D(Gi)-mCherry* (Krashes et al., 2011), the virus was injected at two sites into the right dorsal horn (RC, 0/−0.5 mm; ML, 0.3 mm; DV, 0.6 mm), with the eye of the needle pointing lateral. To prevent leakage and withdrawal of virus, the needle was left in the injection site for 5 min. When the injection was completed, the spine was detached from the clamp, and the connective tissue and skin were sutured and cleaned with sterile saline before the mice were removed from the breathing mask and administered subcutaneously with Buprenorphine (Vetergesic Vet, Orion Pharma, 0.05 mg/kg). The mice were subsequently placed on a heating pad in their cages to wake up. The mice were subjected to behavioral experiments or killed for tissue analyses after a minimum of 2–4 weeks to allow sufficient expression of viral genes.

**Table 1.** Key resources table

Viral vectors and serotypes					
	Vector	Source	Company	Lot and titer	Injection
*AAV8.hSyn-DIO-hM3D(Gq)-mCherry*	pAAV-hSyn-DIO-hM3D(Gq)-mCherry	The vector was a gift from Bryan Roth (Krashes et al., 2011; Addgene viral prep #44361-AAV8; http://n2t.net/addgene:44361; RRID:Addgene_44361)	Addgene	First lot#: v27924 with titer: 2.2 × 10^13^ GC/ml; second lot # v78582 with titer: 2.1 × 10^13 ^GC/ml	One site
*AAV8.hSyn-DIO-hM4D(Gi)-mCherry*	pAAV-hSyn-hM4D(Gi)-mCherry	The vector was a gift from Bryan Roth (Krashes et al., 2011; Addgene viral prep #44362-AAV8; http://n2t.net/addgene:44362; RRID:Addgene_44362)	Addgene	v86749 with titer: 1.8 × 10^13^ GC/ml	Two sites (unilateral)
*AAV8.hSyn-DIO-mCherry*	pAAV-hSyn-DIO-mCherry	The vector was a gift from Bryan Roth (Addgene viral prep # 50459-AAV8; http://n2t.net/addgene:50459; RRID:Addgene_50459)	Addgene	v61605 with titer: 2.2 × 10^13^ GC/ml	One or two sites (unilateral)
*AAVDJ.EF1a-DIO-HTB*	pAAV.EF1a-DIO-HTB	The vector was a gift from Edward Callaway (Addgene plasmid # 44187; http://n2t.net/addgene:44187; RRID:Addgene_44187). The vector was packaged into AAVDJ by Salk institute GT3 (Gene Transfer, Targeting, and Therapeutics) core facility (provided by John Naughton) with funding from NIH-NCI CCSG: P30 014195, an NINDS R24 Core Grant and funding from NEI	Salk Institute	Lot date: 20/12-2018 with titer: 1.09 × 10^12^ VG/ml	Two sites (bilateral)
*AAV8.Syn-flex-TVA.E66T-P2A-oG-WPRE3*	pAAV.Syn-flex-TVA.E66T-P2A-oG-WPRE3	Charité, with technical assistance from Salk investigator John Naughton	Charité	BA-229a with titer: 3.98 × 10^12^ VG/ml	One site
*BRVenvA-1o Rabies Virus, pseudotyped EnvA, mCherry*	pSADB19dG-mCherry	Charité, the material was originally provided by Edward Callaway and distributed through Addgene (plasmid #32630, #32631, #32632, #32633, #32634; Osakada et al., 2011)	Charité	Unknown, with titer: 1.00 × 10^8^ particles/ml	One site
**Antibodies**					
Antibody name	Host animal	Dilution	Company	Catalog number	Lot
NEUN	Mouse	1:500–1:1,000	Millipore	MAB377	
PKCγ	Rabbit polyclonal	1:500	Santa Cruz Biotechnology	sc-211	
IB4		1:500–1:1,000	Invitrogen	I32450	
PAX2	Rabbit polyclonal	1:500	Covance/BioLegend	Poly19010	
GFP	Chicken	1:1,000	Aves Labs	GFP-1020	
NF200	Rabbit	1:1,000	Sigma-Aldrich	N4142	
TRKA	Rabbit monoclonal	1:1,000	Abcam	ab8871	
CGRP	Rabbit polyclonal	1:1,000	Peninsula Laboratories	T-4239.0050	
TH	Rabbit	1:1,000	Millipore	AB152	
SST	Rabbit polyclonal	1:500	Invitrogen	XJ371918	PA5-85759
Anti-rabbit 488	Goat	1:500	Jackson ImmunoResearch	111-095-144	
Anti-rabbit 647	Donkey	1:200 (SST assay); 1:500	Invitrogen	A31573	
Anti-mouse 488	Donkey	1:200	Abcam	ab150105	
Anti-mouse 647	Donkey	1:200	Invitrogen	A31571	
Anti-chicken 488	Donkey	1:500	Invitrogen	SA1-72000	
**RNAscope probes**					
Probe name		Dilution	Company	Catalog number	Channel
*fos*		1:50	Advanced Cell Diagnostics	31692	C1
*Glra3*		1:50	Advanced Cell Diagnostics	490591	C2
*mCherry*		1:50	Advanced Cell Diagnostics	431201	C2
*Nppb*		1:50	Advanced Cell Diagnostics	425021	C1
*Mrgpra3*		1:50	Advanced Cell Diagnostics	548161	C3
*Mrgprd*		1:50	Advanced Cell Diagnostics	417921	C1
*Trpm8*		1:50	Advanced Cell Diagnostics	420451	C3
*Trpv1*		1:50	Advanced Cell Diagnostics	313331	C1
*Vglut2*		1:50	Advanced Cell Diagnostics	319171	C3
*Viaat*		1:50	Advanced Cell Diagnostics	319191	C3

Adult *Glra3*-Cre mice (7 + 7, 7 females, 7 males, 6–26 weeks old) for the Randall–Selitto test were injected with *AAV8.hSyn-DIO-hM4D(Gi)-mCherry* (Krashes et al., 2011) or *AAV8.hSyn-DIO-mCherry* between L1/L2 to target the sacral 2 (S2) segment, affecting the tail (Bennett et al., 1999). The virus was injected into the dorsal horn at two sites with the eye of the needle directing laterally (RC, 0/−0.5 mm; ML, 0.25 mm; DV, 0.45 mm). The mice were assessed to the Randall–Selitto test 2–3 weeks after injection.

The injections for monosynaptic retrograde tracing of adult *Glra3*-Cre mice (*Glra3*-Cre(+): 5 females, 5 males, 7–15 weeks old; *Glra3*-Cre(−): 3 females, 3 males, 7–17 weeks old) were conducted in the same manner as described above for the *AAV8.hSyn-DIO-hM3D(Gq)-mCherry* experiments. The mice were initially injected with helper virus (herein abbreviated as AAV8.Syn-flex-TVA-oG-GFP). To allow sufficient expression of the helper vector genes required for rabies virus host cell entry, the mice were injected with pseudotyped rabies virus *BRVenvA-1o Rabies Virus, pseudotyped EnvA, mCherry* (please see Table 1 for detailed information) 7–8 d after the helper virus injection. One week after the final injection, the mice were sacrificed.

### Immunohistochemistry tissue preparation of developmental and adult virally labeled Glra3-Cre(+) mice

Adult *Glra3*-Cre;*tdTomato* mice (4 females, 7–23 weeks old) and virally spinal cord injected *Glra3*-Cre.mCherry mice (2 females, 2 males, 17–25 weeks old, from the behavioral experiments) were anesthetized in isoflurane (FORANE, Baxter). All mice were subsequently injected intraperitoneally with 0.6 ml (1:1) ketamine (Ketalar, 10 mg/ml, Pfizer) and medetomidine (Domitor, 1 mg/ml, Orion Pharma). When fully anesthetized, the mice were perfused through the left ventricle with 1× PBS, followed by 4% formaldehyde (FA; Histolab). The spinal columns were isolated and placed in 1× PBS, followed by dissection of the tissue area of interest (spinal cord and DRG). The tissues were placed in 4% FA (Histolab) at 4°C overnight. The spinal cords and DRG were dehydrated for 24 h in 15% sucrose and then for 24 h in 30% sucrose for cryoprotection. The tissues were thereafter embedded in optimal cutting temperature (OCT) medium (Bio-Optica) and snap-frozen on dry ice in −80°C isopentane (Sigma-Aldrich), at which temperature the tissues were stored until sectioning. The spinal cords and DRG were sectioned into 16–18 µm sections using a cryostat (Leica Cryocut 1800, Leica), and the sections were collected on glass slides (Superfrost Plus, Thermo Fisher Scientific) as a series of six slides/series. The completed slides were stored at −80°C until further immunohistochemical analyses were performed.

In the tracing experiment, following brain dissections, the brains were fixated in 4% FA (Histolab) at 4°C overnight and thereafter stored in 1× PBS at 4°C until vibratome sectioning. Upon sectioning, the brains were superficially and unilaterally cut with a razor blade to keep track of orientation and subsequently mounted in 4% agarose. The brains were sliced into 70 µm sections (Leica VT1000S, Leica), which were collected into wells as series of five wells/series with five sections/well. All sections were examined for traced mCherry(+) cells using a fluorescent stereomicroscope (Leica MZ16F, Leica). For documentation, the brain sections of one well/series (every fifth brain section) were mounted and embedded in Anti-Fade Fluorescence Mounting Medium (Abcam) on glass slides and covered with glass cover slides (Menzel-Gläser) for imaging.

### Immunohistochemistry

All slides to be used for immunohistochemistry (*Glra3*-Cre;*tdTomato*: 4 females, 7–23 weeks old; *Glra3*-Cre.mCherry: 4 mice; 2 females, 2 males, 17–25 weeks old; traced *Glra3*-Cre(+): 4 females, 2 males, 9–17 weeks old) were placed at RT for 30 min to thaw and dry before initializing the protocols. The assays were either PBS- (NEUN, PKCγ, IB4, NF200, TRKA, CGRP, TH) or TBS- (PAX2, SST) based. In all assays, the sections were washed with 1× PBS/TBS for 4× 10 min before and after the primary antibody incubation. Prior to the primary antibody incubation, the sections were blocked with either supermix [0.25% gelatin and 0.5% Triton X-100 in 1× PBS/TBS (NEUN, NF200, TRKA, CGRP, TH)] or blocking solution [5% donkey or goat serum in 1× PBS/TBS (PKCγ, IB4, PAX2, SST)] for 1 h at RT. In the same solutions [supermix: (PKCγ, IB4, PAX2, SST); blocking solution: (NEUN, NF200, TRKA, CGRP, TH)], the primary and secondary antibodies were incubated, in which the primary antibodies were incubated for 48 h at 4°C and the secondary antibodies with 200 nM/ml DAPI for 2 h at RT. As the final step, the sections were washed 4× 10 min in 1× PBST/TBST (0.1% Tween 20 in 1× PBS/TBS). After completion of protocols, the slides were embedded in Anti-Fade Fluorescence Mounting Medium (Abcam) and covered with glass slides (Menzel-Gläser). The slides were left at 4°C to dry and were stored at this temperature until imaging. For antibody specifics, see Table 1.

### In situ hybridization tissue preparation

Adult *Glra3*-Cre(+) mice microinjected with AAVDJ.EF1a-DIO-HTB (3 females, 2 males, 7–11 weeks) and mice included in the retrograde rabies tracing (1 female and 2 males, 14 weeks) in the L5/L6 spinal dorsal horn were subjected to similar procedures as described previously (Freitag et al., 2021). The HTB protein is a histone-tagged GFP, and this virus was used since the fluorescence could be detected after RNAscope protocol. In brief; 14 d postviral injection, the mice were anesthetized in isoflurane (FORANE, Baxter), followed by intraperitoneal injection of 0.6 ml (1:1) ketamine (Ketalar, 10 mg/ml, Pfizer) and medetomidine (Domitor, 1 mg/ml, Orion Pharma). To minimize the risk of contamination and altered gene expression, the mice were perfused in autoclaved ice-cold 1× PBS. In the same solution, the spinal columns were quickly dissected, and the spinal area containing the viral fluorescence was isolated. The tissues were immediately embedded in OCT medium (Bio-Optica) and snap-frozen on dry ice in −80°C isopentane (Sigma-Aldrich), at which temperature the tissues were stored until sectioning. The tissues were cryosectioned (Leica Cryocut 1800, Leica) into 12–14 µm sections and were collected onto Superfrost Plus (Thermo Fisher Scientific) glass slides as series consisting of six slides with 7–8 sections/slide for the *Glra3*-Cre(+) AAVDJ.EF1a-DIO-HTB injected mice and eight slides with 3 sections/slide for the sensory stimulated C57BL/6J mice. To prevent mRNA degradation and contamination, the completed series were stored at −21°C until sectioning was finished. The slides were stored at −80°C until the RNAscope Fluorescent Multiplex kit [Advanced Cell Diagnostics (ACD), catalog #320850] protocol commenced.

### Fluorescent in situ hybridization

The fluorescent in situ hybridization was performed using the RNAscope Fluorescent Multiplex kit (ACD, catalog #320850) in accordance with ACD guidelines for fresh frozen tissues, with minor modifications (Wang et al., 2012) on sections from *Glra3*-Cre.HTB and sensory stimulated C57BL/6J mice. In brief, as performed previously (Freitag et al., 2021), the slides to be used were taken from −80°C and immediately fixated in 4% FA (Histolab) for 15 min at RT before being washed in autoclaved 1× PBS for 2 min. The tissues were thereafter dehydrated in a stepwise increase of EtOH concentration; 3 min in 50%, 3 min in 70%, and 2× 5 min in 100% (Merck KGaA). The slides were placed at RT for 5 min to dry, whereafter a hydrophobic barrier was made around the chosen sections (three sections/mouse), using an ImmEdge pen (Vector Laboratories). The sections were incubated in protease IV for 30–40 min at RT, followed by 3× 5 min washing in autoclaved 1× PBS. The sections were incubated in target probes (for specifics see Table 1) 1:50 in probe diluent (ACD, catalog #300041) for 2 h at 40°C in a hybridization oven (HybEZ II Oven, ACD). The following amplification steps were performed at 40°C in the hybridization oven, and the sections were washed 2× 2 min in RT washing buffer between each amplification step: AMP 1-FL for 30 min, AMP 2-FL for 15 min, AMP 3-FL for 30 min, and AMP 4-FL for 15 min. The coloring step using AMP 4-FL was performed to enable the combination with the viral fluorescence. Lastly, the slides were washed 2× 2 min in washing buffer before 30 s incubations in DAPI and mounting in Anti-Fade Fluorescence Mounting Medium (Abcam). The slides were covered with glass slides (Menzel-Gläser) and were left at 4°C to dry. The slides were stored at this temperature until imaging.

### Image acquisition and quantification

Images of immunohistochemistry treated sections were acquired using a wide-field Olympus BX61WI fluorescence microscope (Olympus) with a 10× objective, for which the brightness and contrast were optimized for each channel during image acquisition and quantification. The RNAscope treated sections were acquired with wide-field 20× magnification with an Olympus BX61WI fluorescence microscope (Olympus) or an Axio Imager.Z2 (ZEISS), where each channel was set to be automatically optimized for each image, but had to be further optimized during image analysis. Here, the optimal intensity and contrast was set for one image (reference image) and the settings of the other images were set to match the reference image. The images were manually quantified using the Fiji (ImageJ 1.52f) Cell Counter plug-in.

#### Immunohistochemistry spinal cord

All *Glra3*-Cre.*tdTomato* (2 females, *n* sections/mouse: 3) or *Glra3*-Cre.mCherry (2 females and 2 males, *n* sections/mouse: 3–5) mice with DAPI overlap were quantified depending on layer location (IB4, outer lamina II; PKCγ, inner lamina II) and marker protein (NEUN) coexpression*.*

#### Immunohistochemistry retrograde rabies tracing, spinal cord

*Glra3*-Cre(+) mice: A DAPI cell with overlap of helper virus GFP and rabies virus mCherry was considered a starter cell, and a DAPI cell with only mCherry overlap was considered a presynaptic traced cell. The coexpression of starter and traced cells was quantified for NEUN and PAX2 (5 females, 5 males, *n* section/mouse/assay: 2–11). *Glra3*-Cre(−) mice: The overlap of helper virus GFP and rabies virus mCherry with DAPI overlap was quantified (3 females, 3 males, every sixth section analyzed).

#### Immunohistochemistry retrograde rabies tracing, DRG

The overlap of traced mCherry DAPI+ cells with NF200, TRKA, CGRP, IB4, TH, and SST were quantified (NF200: 2 females, 2 males, *n* sections/mice: 2–5; TRKA: 2 females, 2 males, *n* section/mice: 1–8; CGRP: 1 female, 2 males, *n* sections/mice: 2–5; IB4: 3 females. 1 male, *n* sections/mice: 2–5; TH: 4 females, 2 males, *n* sections/mice: 1–7; SST: 2 females, 1 male, *n* sections/mice: 3–5).

#### Brain scanning for mCherry(+) traced cells in Glra3-Cre(+) and Glra3-Cre(−) mice

Whole-brain section images were acquired in the mCherry (500 ms) and wide-field black and white (15% light source intensity, 5 ms) channels of every fifth section, using tiles (ZEISS) to scan for mCherry(+) traced cells (*Glra3*-Cre(+): 5 females, 5 males; *Glra3*-Cre(−): 3 females, 3 males).

#### RNAscope, Glra3-Cre.HTB

All *Glra3*-Cre.HTB cells with DAPI overlap were considered cells and one read of the targeted probe could be visualized as one dot. A *Glra3*-Cre.HTB cell was considered to be expressing the targeted gene (*Glra3*, *Vglut2*, or *Viaat*) if the overlapping #dots ≥3 (3 females and 2 males, *n* sections/mouse: 2–4). One section from the *Glra3/Viaat* assay was excluded due to weak signal from both probes.

#### RNAscope, retrograde rabies tracing DRG

DAPI cell was considered expressing the targeted gene (*mCherry*, *Nppb*, *Mrgpra3*, *Mrgprd*, *Trpv1*, *Trpm8*) if the #dots ≥3 (*Nppb*: 1 female and 1 male, *n* sections/mice:4–6; *Mrgprd* and *Mrgpra3*: 1 female and 1 male, *n* sections/mice:4–6, *Trpv1* and *Trpm8*: 2 females and 1 male, *n* sections/mice:2–6).

#### RNAscope, *fos* expression in Glra3 expressing cells following sensory stimulation

The experimenter was blinded to the treatment received by the mouse and the *Vglut2*/*Viaat* probes, so no randomization was needed in the quantification. A DAPI cell was considered to express the targeted gene (*Glra3*, *fos*, and *Vglut2* or *Viaat*) if the #dots ≥3 and #dots ≥5 for *fos* (three mice/stimulus, *n* sections/mouse: 3). One section from the scratch analysis was excluded due to poor tissue quality. To obtain a high resolution, two images of each dorsal horn were acquired and later merged together using Adobe Photoshop 22.3 to a composited representative image of the dorsal horn. The result is presented as percentage ± SEM.

### Electrophysiology

For patch-clamp recordings, spinal cord transverse slices were made from *Glra3*-Cre;*tdTomato* mice (13 females, 11 males, 4–35 weeks old) according to a previously described protocol (Freitag et al., 2019). For root stimulations, the spinal cord was cut at a 60° angle and the slice thickness was increased to 400 µm in order to get transverse slices with attached dorsal roots. After incubation, the slice was transferred to a recording chamber, where *Glra3*-Cre;*tdTomato* neurons were visualized via a fluorescent LED light source (CoolLED system) on a Prime BSI Express Scientific sCMOS camera (Teledyne Photometrics) through 60× or 10× water-immersion objectives [LUMPlan FI, 0.90 numerical aperture (NA), Olympus]. Borosilicate glass capillaries (GC150F-10 Harvard Apparatus) were used to pull patch electrodes (6–10 MΩ) with a flaming/brown micropipette puller (P-1000, Sutter Instrument). The following is the internal solution of patch pipettes (in mM): 130 K-gluconate, 40 HEPES, 1.02 MgCl_2_, 2.17 MgATP, 0.34 NaGTP, with pH adjusted to 7.2 using 1 M KOH. Liquid junction potential was corrected before each recording. Whole-cell patch-clamp signals were amplified with a MultiClamp 700B amplifier (Molecular Devices), digitalized at 20 kHz with Digidata 1440A (Molecular Devices), low-pass filtered at 10 kHz, and acquired in WinWCP software (Dr. J. Dempster, University of Strathclyde).

When the whole-cell configuration was achieved, action potentials (APs) were induced, in the current-clamp mode via current steps from 0 to 150 pA with increments of 10 pA (pulse duration, 500 ms), to monitor the viability and the firing pattern of the patched neuron. The rheobase was determined by using 1 pA increment current steps (pulse duration, 500 ms). The neuron was then held at −60 mV in the voltage-clamp mode. When a stable baseline was achieved in a continuous voltage-clamp recording, 300 µM glycine was applied through the perfusion system to the recording chamber to verify the expression of GLRs on the patched neuron. The hyperpolarization was then blocked by 10 µM strychnine to further confirm that the response was due to the expression of GLRs.

In root stimulation experiments, the dorsal root was identified using the 10× objective and sucked into a suction pipette. The stimulating electric pules were applied via the suction pipette from an A365 Stimulus Isolator (World Precision Instruments). Stimulation pulses with a duration of 0.2 ms were used for activation of the dorsal root, while in some cases 0.5 ms pulse durations were used to activate the C-fiber. The transduction velocities of different afferent fibers were used to determine monosynaptic inputs (Pan et al., 2019), which were further confirmed by none failure responses with consistent onset latencies, where patched cells responded to a minimum of 10 consecutive root stimulations at 1 Hz and the latency variation was <1 ms (Pinto et al., 2008; Pan et al., 2019). Data analyses were done by Clampfit 10.3 (Molecular Devices), Mini Analysis (Synaptosoft), and GraphPad Prism (GraphPad Software). No neurons were excluded in the postanalysis.

### Cell filling

Neurobiotin Tracer (Vector Laboratories) was added into the intracellular solution (4 mg/ml) and diffused into the target *Glra3*-Cre;*tdTomato* cells during the patch-clamp recording. The diffusion of Neurobiotin was further assisted by injecting depolarizing current pulses (0.2–0.5 nA; duration, 150 ms) into the cell at 2 Hz for 10–15 min. After the filling, the patch pipette was carefully detached from the cell and removed from the recording chamber. The excessive Neurobiotin in the tissue was removed by perfusing the slice for at least 15 more min after the removal of the pipette. The slice was then transported into an Eppendorf tube and fixed in 4% FA (Histolab) overnight at 4°C. Fixed slices were washed with 1× PBS (Fisher BioReagents) 4x 10 min before the staining. Slices were stained for PKCγ using the same procedure described in previous immunohistochemistry section. Additionally, streptavidin Alexa Flour 488 conjugate (Invitrogen) was added to the primary antibody staining solution with 1:1,000 dilution ratio for Neurobiotin staining. The mounted slice was imaged using a ZEISS LSM700 confocal microscope (ZEISS) with 10× and 20× objectives. The morphology of a filled neuron was reconstructed using the Simple Neurite Tracer plug-in in the NIH ImageJ software (National Institutes of Health).

### Basal behavioral observation after chemogenetic activation or inhibition of Glra3-Cre(+) neurons

*Glra3*-Cre(+) mice (*Glra3*-Cre.hM3Dq and *Glra3*-Cre.mCherry: 7 + 8 mice, 7 females and 8 males; *Glra3*-Cre.hM4Di and *Glra3*-Cre.mCherry: 8 + 8 mice, 11 females, 5 males) unilaterally injected in L5 with AAV8.hsyn-DIO-hM3D(Gq)-mCherry, AAV8.hsyn-DIO-hM4D(Gi)-mCherry, or AAV8.hsyn-DIO-mCherry were acclimatized to a plastic cylinder arena (diameter, 19 cm; height, 29 cm; surface area, 283 cm^2^) with a mirror to obtain a 360° view for 20 min. The mice were injected intraperitoneally with 0.1 mg/kg of freshly prepared clozapine *N*-oxide (CNO; AK Scientific, 0.02 mg/ml dissolved in 0.02% DMSO in sterile saline). The basal behavior of the mice following CNO administration was recorded for 30 min (for *Glra3*-Cre.hM3Dq recordings) or 60 min (for *Glra3*-Cre.hM4Di recordings). The duration and frequency of targeted behaviors were analyzed for the total recording time. The same experimenter scored all the behavior recordings and was blinded for the viral vectors used during the experiments. The licking/biting of the ipsilateral paw were scored as one behavior, for which the episodes were scored when contact between the paw and face could be clearly visualized. The guarding and stomping behaviors were also scored. Guarding was defined as the time the mouse spent sitting still with its paw in the air. Stomping was interpreted as a mouse rapidly lifting and lowering the hindpaw while being either still or in movement. No mice were excluded from the analysis

### Injections of saline or pruritogens

Two days prior to the stimulus recording, the right calves of the mice were shaved and cleaned with sterile saline. Adult *Glra3*-Cre(+) mice injected with AAV8.hsyn-DIO-hM4D(Gi)-mCherry or control AAV8.hsyn-DIO-mCherry were injected with 0.1 mg/kg freshly prepared CNO (AK Scientific, 0.02 mg/ml dissolved in 0.02% DMSO in sterile saline) and thereafter returned to their respective home cages. After 30 min, the mice were placed in a plastic cylinder arena with a mirror to obtain a 360° view for 10 min to acclimatize to the setup. The mice were subsequently injected subcutaneously in the dorsolateral calf with either 10 µl of saline (8 + 8 mice; 9 females, 7 males), 20 µg compound 48/80 (Sigma-Aldrich, catalog #c2313, dissolved in sterile saline; 8 + 8 mice; 8 females, 8 males), or 10 mM chloroquine phosphate (Sigma-Aldrich, catalog #PHR1258, dissolved in sterile saline, 8 + 8 mice; 9 females, 7 males). The mice were returned to the plastic cylinder area and recorded for 30 min. Licking of the calf is indicative of pain, while biting demonstrates itch (LaMotte et al., 2011). However, since we had difficulties separating these behaviors while scoring, the total duration and frequency of licking/biting toward the injected calf was scored as one behavior. These episodes were scored when contact between the calf and face could be clearly visualized. No mice were excluded from the analysis.

### Randall–Selitto test

Two days prior to the experiment, a plastic cylinder (Model 84, IITC Life Science) was placed in each home cage to acclimatize the mice to the setup. Adult *Glra3*-Cre(+) mice (7 + 7 mice; 7 females, 7 males) injected with either AAV8.hsyn-DIO-hM4D(Gi)-mCherry or AAV8.hsyn-DIO-mCherry between L1/L2 were intraperitoneally administered 0.1 mg/kg freshly prepared CNO (AK Scientific, 0.02 mg/ml dissolved in 0.02% DMSO in sterile saline) and thereafter returned to their respective home cages. Ten minutes later, the mice were allowed to enter the plastic cylinder and were placed in the Randall–Selitto setup (Analgesy-meter, UGO Basile) for ∼30 min. When 40 min had passed since the CNO injection, the mechanical threshold (g), at which pressure the mouse retracted its tail, was measured twice per mouse at different locations on the tail with at least 5 min between the measurements. One female and one male injected with AAV8.hsyn-DIO-hM4D(Gi)-mCherry were excluded from the analysis due to lack of mCherry expression in the post hoc verification step.

### Hargreaves test

Adult *Glra3*-Cre(+) mice (8 + 8 mice; 11 females, 5 males) injected with AAV8.hsyn-DIO-hM4D(Gi)-mCherry or control AAV8.hsyn-DIO-mCherry were initially acclimatized for 60 min in the Hargreaves setup (transparent acrylic glass chambers on glass floor). Baseline thermal sensitivity was measured by directing the Hargreaves heat source (IITC Life Science), guided by a light pointer, to the plantar surface of the right hindpaw, for which the time from turning on the thermal source until the mouse withdrew/flinched its paw was noted. The cutoff time was set to 20 s to avoid tissue damage, and the withdrawal time was measured twice with at least 5 min intervals in between each measurement. After completed measurements, the mice were injected intraperitoneally with 0.1 mg/kg freshly prepared CNO (AK Scientific, 0.02 mg/ml dissolved in 0.02% DMSO in sterile saline) and placed back into the Hargreaves setup. Forty minutes after the CNO administration, the withdrawal time measurement was repeated. No mice were excluded from the analysis.

### Acetone drop test

Adult *Glra3*-Cre(+) mice (8 + 8 mice; 8 females, 8 males) injected with either AAV8.hsyn-DIO-hM4D(Gi)-mCherry or AAV8.hsyn-DIO-mCherry were allowed 60 min acclimatization to the gridded surface. Forty minutes before the first measurement, the mice were injected intraperitoneally with 0.1 mg/kg freshly prepared CNO (AK Scientific, 0.02 mg/ml dissolved in 0.02% DMSO in sterile saline), and returned to the setup. The mice were subjected to a drop of acetone solution (9:1 acetone in water, Labscan) on the plantar surface of the right hindpaw, where the total duration of sensory aversive behaviors, including lifting, flinching, and licking/biting of the paw, was recorded. The stimulation was performed twice with at least 5 min intervals in between each application of the acetone solution. No mice were excluded from the analysis.

### Sensory stimulation for *fos* detection

#### Pruritic stimulation of urethane-anesthetized mice

To detect activation of *Glra3*-expressing cells following sensory stimulation, adult C57BL/6J mice (10–14 weeks old, 3 mice/stimulus, 15 mice in total) were initially anesthetized with 2 g/kg urethane (Sigma-Aldrich, catalog #U2500, 125 mg/ml in sterile saline) through intraperitoneal injection to minimize neuronal activity caused by prurito- and nocifensive behavior. To prevent eye damage and dehydration, Oftagel (Santen Oy) was applied to eyes, and the mouse was injected subcutaneously with 0.5 ml saline. To maintain body temperature, a glove filled with body temperature water, which was continuously replaced to sustain temperature, was placed next to the mouse. When the mouse had been fully anesthetized for 10 min, the mouse was subjected to the stimulus. For pruritic stimulations, the mice were injected subcutaneously into the right dorsolateral calf either with 10 µl saline (1 female and 2 males) or a pruritic substance: 20 µg compound 48/80 (Sigma-Aldrich, catalog #c2313, dissolved in sterile saline, 1 female and 2 males) or 20 mM chloroquine (Sigma-Aldrich, catalog #PHR1258, dissolved in sterile saline, 1 female and 2 males).

#### Noxious mechanical stimulation of urethane-anesthetized mice

The mouse was either subjected to pinching (1 female and 2 males) or scratching (2 females and 1 male) of the skin on the right dorsolateral calf. The pinching was performed 5× for 5 s using tweezers, with 5 s resting periods in between each pinching episode. The scratching was conducted for 30 s with 2 Hz and ∼300 mN (30.6 g), using an artificial mouse claw in scratch position. Forty minutes after application of the stimulus, the mouse was injected intraperitoneally with 0.05 ml ketamine (Ketalar, 10 mg/ml, Pfizer) and 0.05 ml medetomidine (Domitor, 1 mg/ml, Orion Pharma), followed by perfusion and tissue preparation for RNAscope, as described above.

#### Hargreaves stimulation for *fos* detection in awake mice

Adult C57BL/6J mice (2 females and 1 male, 11–17 weeks old) were subjected to the same Hargreaves protocol as described above for baseline measurements. After completed stimulation, 40 min were allowed to pass until the mouse was injected intraperitoneally with 0.7–0.8 ml ketamine (Ketalar, 10 mg/ml, Pfizer) and medetomidine (Domitor, 1 mg/ml, Orion Pharma; 1:1), followed by perfusion and tissue preparation for RNAscope, as described above. Same mice but separate sections have been used in a manuscript under revision. No mice were excluded from the analysis.

### Experimental design and statistical analyses

All behavioral testing was performed a minimum of 2–4 weeks after viral injection to allow sufficient expression of viral vector genes. *Glra3*-Cre.hM3Dq mice were only included in one basal behavioral analysis/mouse, except for 2 (*Glra3*-Cre.hM3Dq) + 3 (*Glra3*-Cre.mCherry) mice that also were subjected to an initial analysis (one CNO injection; data not shown) a few weeks prior to establish an optimal CNO concentration. *Glra3*-Cre.hM4Di mice were included in maximum four behavioral tests (including basal recording) with a minimum of 1 week between the tests. The basal recording was conducted first and the following tests were not conducted in a specific order. *Glra3*-Cre.mCherry mice were included in maximum four behavioral tests (including basal recording) with a minimum of 1 week between the tests. The tests were not conducted in a specific order. The mice were returned to their home cages after each completed behavioral test. No mice were excluded from the behavioral analyses presented. No randomization was used. Mice were arbitrary assigned to different treatments (e.g., injections of viral vectors) based on sex. All the behavior experiments were conducted by the same female experimenter, who was blinded to viral vectors (control vs chemogenetic). In the acetone and Randall–Selitto tests, an additional female experimenter was conducting the experiment (also blinded to the viral vector injected), so no randomization was needed or possible. Reporter expression was validated and documented in all mice after chemogenetic behavioral testing to ensure presence of DREADD (designer receptors exclusively activated by designer drugs) or control vector at the correct spinal segments. The experimental groups were matched to the best extent in terms of sex and littermates. In the sensory stimulation tests to examine *fos*, the mice were arbitrary assigned to the different stimuli, but we ensured that both sexes were used in the testing.

The number of mice per behavioral and in situ experiment was not based on any statistical calculations prior to the experiments. Sample sizes are in line with similar studies in the field (Bourane et al., 2015; Foster et al., 2015; Häring et al., 2018). All data were analyzed in GraphPad Prism (version 9 or 10). The normal distribution of the mean data per mouse was analyzed using the Shapiro–Wilk normality test (*α *= 0.05). To compare mean values, we performed either a two-tailed Student’s *t* test or Mann–Whitney *U* test. In the basal hM3Dq experiment, for which the mean value of the control mCherry groups was zero for stomping and guarding behaviors, a chi-square test was performed to compare the mean values between these groups. In order to compare the mean values between multiple parameters (viral vector and pre/post CNO injection) in the Hargreaves test and to compare the differences in the number of the targeted cells following saline, compound 48/80, and chloroquine injections, a one-way ANOVA with Šídák's multiple-comparisons test was used. The results are presented as mean ± SEM.

## Results

### *Glra3-Cre;tdTomato* neurons are predominantly located in laminae III–IV and the adult *Glra3*-Cre population consists of a major excitatory and a minor inhibitory population

**Figure 1.**
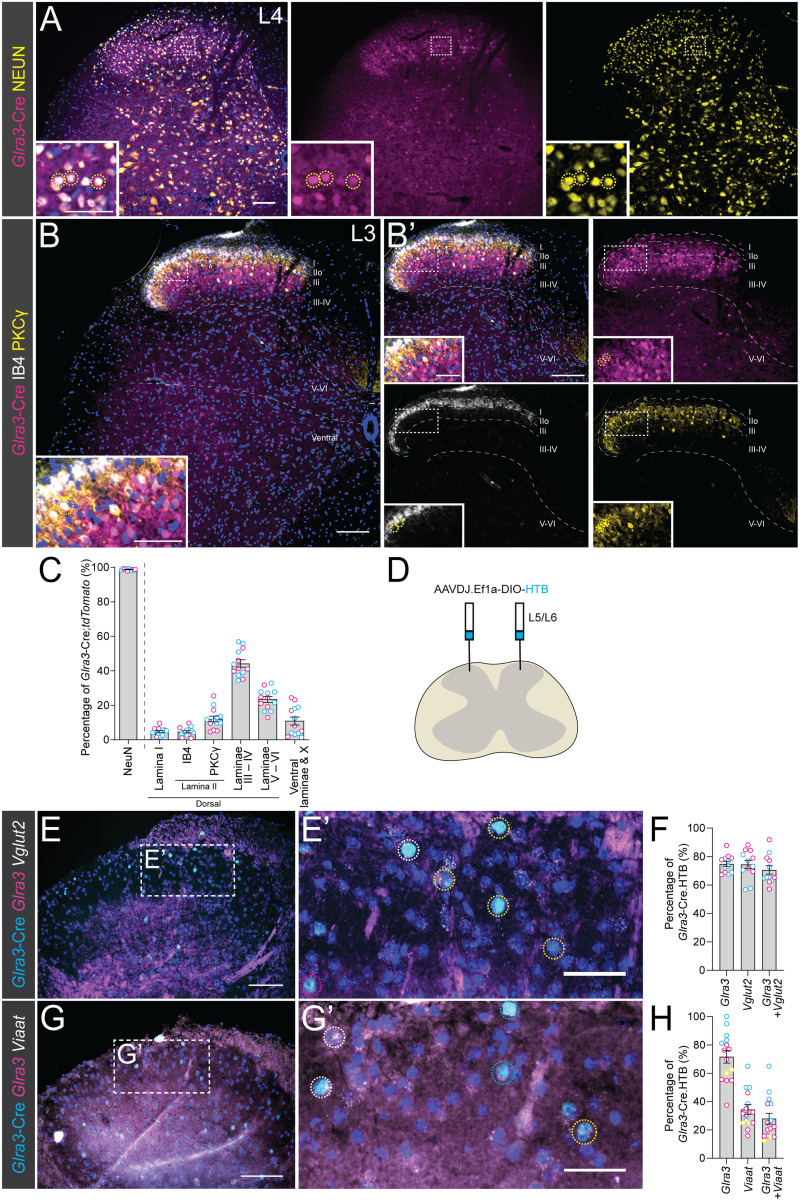
*Glra3*-Cre;*tdTomato* neurons are predominantly located in laminae III–IV and the adult *Glra3*-Cre population consists of a major excitatory and a minor inhibitory population. ***A***, Overlap of spinal lumbar *Glra3*-Cre;*tdTomato* cells (magenta) and neuronal marker NEUN (yellow). Yellow dotted circles display examples of tdTomato(+)NEUN(+) cells. ***B***, ***B’***, Location of tdTomato(+) cells (magenta) in IB4(+) outer lamina II (white), PKCγ(+) inner lamina II (yellow), laminae III–IV, laminae V–VI, and ventral laminae defined from The Spinal Cord atlas (Anderson et al., 2009). Yellow dotted circles represent examples of tdTomato(+)PKCγ(+) cells. ***C***, Scatter bar plot of the occurrence of tdTomato(+)NEUN(+) and tdTomato(+) cells in different spinal areas (2 females, *n* images: NEUN: 10; 2 females, *n* images: PKCγ/IB4: 13). ***D***, Schematic illustration of AAVDJ.Ef1a-DIO-HTB lumbar 5/lumbar 6 (L5/L6) microinjection into *Glra3*-Cre(+) mice. ***E***, ***E’***, Overlap of *Glra3* (magenta) and *Vglut2* (white) in adult *Glra3*-Cre.HTB neurons (cyan). Yellow dotted circles indicate *Glra3*(+)*Vglut2*(+), magenta dotted circles denote *Glra3*(+)*Vglut2*(−), and white circles represent *Glra3*(−)*Vglut2*(+) in *Glra3*-Cre.HTB(+) cells. ***F***, Scatter bar plot of percentages of *Glra3*-Cre.HTB(+) neurons expressing analyzed genes when targeting *Vglut2* (2 males, *n* sections: 6, images: 12; ***G***, ***G’***) *Glra3*-Cre.HTB neurons’ expression of *Glra3* (magenta) and *Viaat* (white). Yellow dotted circles indicate *Glra3*(+)*Viaat*(−), white dotted circles represent *Glra3*(−)*Vglut2*(+), and blue dotted circles show *Glra3*(−)*Vglut2*(−) in *Glra3*-Cre.HTB(+) cells. ***H***, Scatter bar plot displaying percentages of *Glra3*-Cre.HTB(+) neurons expressing analyzed genes when targeting *Viaat* (3 females, *n* sections: 7, images: 15). Scale bars: ***A***, ***B’***, ***E***, ***G***, 100 µm; ***B***, 150 µm; enlargement in ***A***, ***E’***, ***G’***, 50 µm; enlargement in ***B***, 75 µm. The observational dots in the scatter bar plots (***C***, ***F***, ***H***) represent a unilateral part of the spinal cord and the different dot colors signify different mice. Results are presented as mean ± SEM.

First, immunohistochemistry was used to examine the anatomical location and molecular characteristics of the spinal *Glra3*-Cre population using the *tdTomato* reporter line (Fig. 1*A–C*). Immunostaining for the neuronal marker NEUN (Fig. 1*A*) showed that almost all tdTomato(+) cells were neurons (98.7 ± 0.2%, 1,637/1,659). The neurons were most frequently found in the dorsal horn (dorsal horn: laminae I–VI, 89.1 ± 2.3%, 1,528/1,713; ventral horn: laminae VII–X, 10.9 ± 2.3%, 185/1,713), especially in laminae III–IV (44.2 ± 2.1%, 753/1,713) and laminae V–VI (23.4 ± 1.6%, 405/1,713). Smaller tdTomato(+) populations were found in lamina I (4.8 ± 0.6%, 78/1,713), the outer lamina II defined by IB4 staining (Todd, 2017; 4.7 ± 0.7%, 80/1,713), and the inner lamina II defined by PKCγ (Polgár et al., 1999; Peirs et al., 2014; 12.1 ± 1.6%, 212/1,713), in which 52.4 ± 5.2% (117/212) of the cells were tdTomato(+)PKCγ(+) (Fig. 1*B*,*B’*). Collectively, the *Glra3*-Cre;*tdTomato* neurons were located throughout the spinal cord (Fig. 1*C*) and were most commonly found in laminae III–IV.

Single-cell RNA sequencing (scRNAseq) has identified *Glra3* in both excitatory SCGLU10 and Glut9 and in inhibitory Gaba8–9 spinal dorsal horn neuronal clusters among others (Häring et al., 2018; Zeisel et al., 2018). To further examine the molecular characteristics of the *Glra3*-Cre population and to address adult *Glra3*-Cre expression, fluorescent in situ hybridization using the RNAscope approach (Wang et al., 2012) was performed. The said method targeted *Glra3*, the excitatory marker *Vglut2* (*Vesicular glutamate transporter 2*, *Slc17a6*), and the inhibitory marker *Viaat* (*Vesicular inhibitory amino acid transporter, Slc32a1*) in adult AAVDJ.Ef1a-DIO-HTB labeled *Glra3*-Cre(+) neurons (Fig. 1*D–H*). HTB is a histone-tagged protein that was used due to its ability to be detected following the RNAscope protocol. *Glra3* was expressed by 74.8 ± 1.8% (436/571) of the *Glra3*-Cre.HTB(+) cells in the analysis also targeting *Vglut2* and by 71.7 ± 4.3% (235/342) of the *Glra3*-Cre.HTB(+) cells in the analysis also targeting *Viaat*. These findings indicated that the mouse line and Cre-dependent virus mark the *Glra3*(+) population (Fig. 1*E–H*). In the *Vglut2*-examining assay (Fig. 1*E*,*E’*), *Vglut2*(+) and *Glra3*(+)*Vglut2*(+) were found in 74.5 ± 2.8% (437/571) and 70.6 ± 2.8% (318/517) of the *Glra3*-Cre.HTB(+) population, respectively (Fig. 1*F*). Meanwhile, *Viaat*(+) was found in 34.4 ± 3.3% (116/342) and *Glra3*(+)*Viaat*(+) in 28.0 ± 3.75% (89/342) of the *Glra3*-Cre.HTB(+) neurons (Fig. 1*G*,*H*). These results suggest that the adult spinal *Glra3*-Cre population consists of *Glra3*-expressing neurons found in a major *Vglut2*(+) excitatory population and a smaller *Viaat*(+) inhibitory population.

### *Glra3-Cre;tdTomato* neurons respond to glycine and the populations display a heterogeneous firing pattern

**Figure 2.**
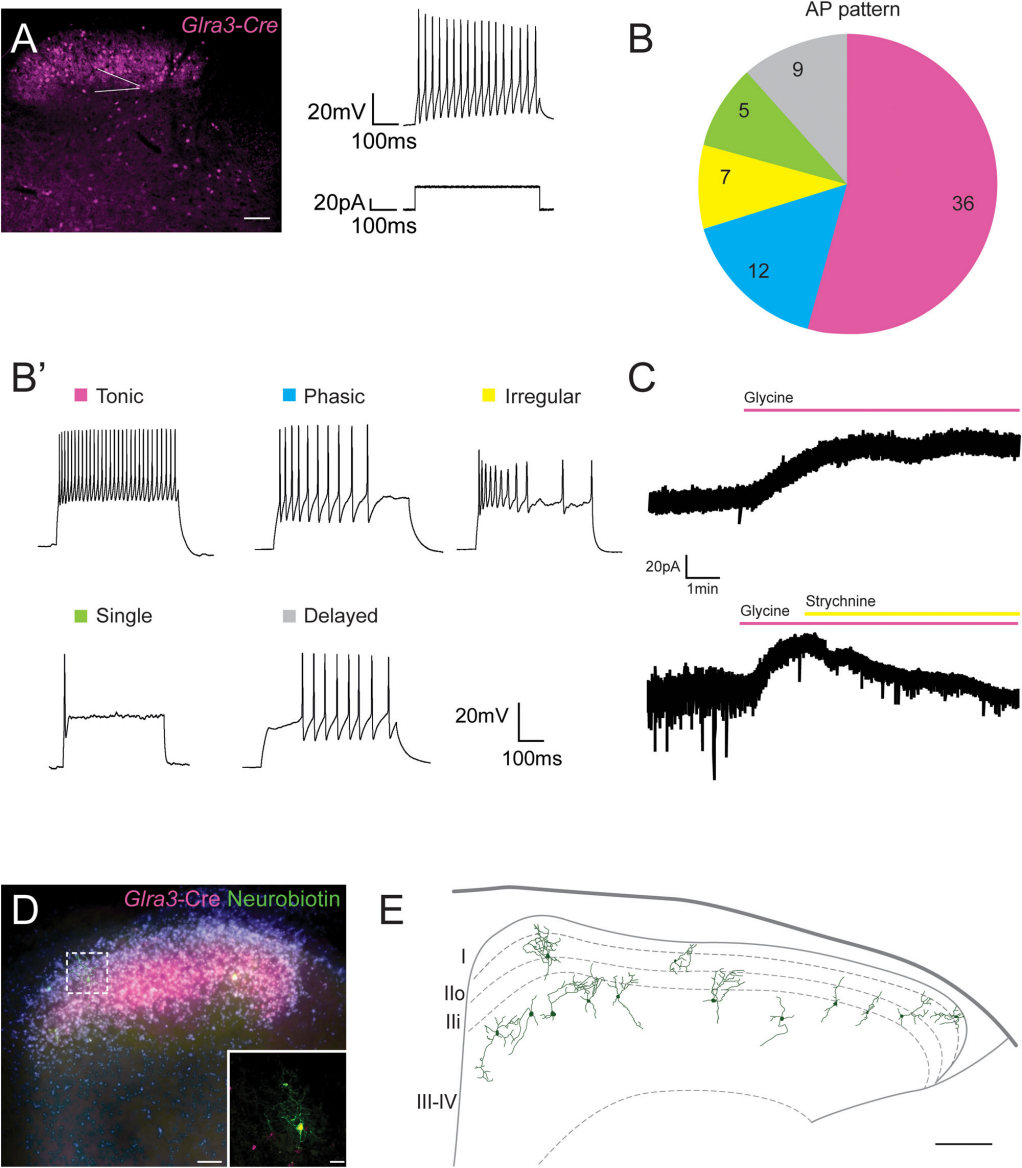
*Glra3*-Cre;*tdTomato* neurons respond to glycine and the populations display a heterogeneous firing pattern. ***A***, Patch-clamp recordings of spinal *Glra3*-Cre;*tdTomato* neurons (magenta). A schematic patch pipette is indicated with a white arrowhead. Scale bar: 50 µm. The image on the right represents a recording of AP firing (above) upon stimulation with a depolarizing current for a duration of 500 ms (below). ***B***, Pie chart of the distribution of different AP firing patterns (8 females, 7 males, *n* cells: 69). ***B’***, Representative recording of each firing pattern. The scale bar applies to all five traces. ***C***, Representative recordings of the hyperpolarizing current induced by glycine (300 µM, *n* cells: 13, above) and blockage by strychnine (10 µM, *n* cells: 6, below). The scale bar applies to both traces. ***D***, A Neurobiotin filled neuron in a mouse spinal cord slice. Magenta(+) cells are *Glra3*-Cre;*tdTomato* neurons, PKCγ staining is presented in white, blue is DAPI staining (scale bar, 50 µm). The zoomed in image shows the Neurobiotin filled neuron stained with Alexa Fluor 488 streptavidin conjugate (in green; scale bar, 20 µm). ***E***, Morphological and locational reconstruction of all Neurobiotin filled neurons (*n* cells: 13; scale bar, 100 µm). Laminae are defined from The Spinal Cord atlas (Anderson et al., 2009).

Patch-clamp recordings were used to examine electrophysiological properties of *Glra3*-Cre(+) neurons. The recorded *Glra3*-Cre;*tdTomato* neurons had an average resting membrane potential of −59.9 ± 1.2 mV, input resistance of 879 ± 70.1 MΩ, and membrane capacitance of 55.1 ± 4.3 pF (Table 2). All recorded neurons fired APs upon electrical stimulation of 500 ms duration and increments of 10 pA (Fig. 2*A*). Moreover, the APs comprised five different firing patterns (Fig. 2*B*,*B’*), with 52% of APs being tonic (36/69), 17% phasic (12/69), 7% single (5/69), 13% delayed (9/69), and 10% irregular (7/69; Fig. 2*B*,*B’*; Table 2). These firing patterns resemble previously identified categories of mouse dorsal horn neurons in terms of AP patterns (Hu and Gereau, 2003, 2011; Heinke et al., 2004). The tdTomato(+) neurons had an average rheobase of 22.4 ± 2.8 pA, an AP threshold of −30.9 ± 1.1 mV, and a peak AP of 21.7 ± 1.8 mV. Inter-group comparison showed that only neurons with delayed AP patterns had lower resting membrane potentials. No differences were observed in any other measured electrophysiological properties among neurons in the five AP pattern categories (Table 2). Collectively, the *Glra3*-Cre populations constitute five categories of neurons according to their firing patterns, with homogenous intrinsic membrane properties. The presence of functional glycine receptors on the recorded neurons was determined by applying glycine to the recording chamber in a voltage-clamp mode, where cells were held at −60 mV. All glycine applied *Glra3*-Cre;*tdTomato* neurons showed hyperpolarizing currents (an average of −34.8 ± 5.7 pA), and the glycine-induced current was completely blocked by the glycine receptor antagonist strychnine (Fig. 2*C*).

**Table 2.** Membrane and firing properties of *Glra3*-Cre:*tdTomato* neurons

Firing pattern	Tonic (*n* = 36)	Delayed (*n* = 9)	Phasic (*n* = 12)	Irregular (*n* = 7)	Single (*n* = 5)	Total (*n* = 69)
Resting membrane potential (mV)	−59.37 ± 1.54	−72.33^a^ ± 3.15	−56.33 ± 2.78	−58.14 ± 1.83	−52.40 ± 3.17	−59.91 ± 1.22
Input resistance (MΩ)	1,010.6 ± 119.1	732.78 ± 123.53	796.33 ± 119.4	718.71 ± 150	649.60 ± 110.31	879.43 ± 70.08
Capacitance (pF)	56.79 ± 7.12	79.22 ± 7.38	48.42 ± 6.84	51.71 ± 7.54	26.40 ± 6.23	55.07 ± 4.34
Rheobase (pA)	21.24 ± 2.82	23.33 ± 3.51	19.00 ± 4.55	20.43 ± 2.89	41.40 ± 12.82	22.56 ± 2.03
AP threshold (mV)	−31.38 ± 1.55	−30.84 ± 2.73	−28.34 ± 2.88	−36.69 ± 1.65	−25.14 ± 6.51	−30.94 ± 1.12
AP rising time (ms)	1.08 ± 0.12	1.35 ± 0.08	1.98 ± 0.35	1.39 ± 0.12	1.27 ± 0.32	1.34 ± 0.10
AP peak (mV)	21.43 ± 2.69	28.43 ± 2.77	20.80 ± 5.15	17.16 ± 5.16	19.59 ± 7.68	21.71 ± 1.85

a Delayed AP group showed lower resting membrane potential compared with all the other groups in two-tailed one-way ANOVA followed by Turkey's multiple-comparisons test.

Two studies have described that GLRA3 is present in the superficial laminae of the dorsal horn (Harvey et al., 2004; Werynska et al., 2021), while a third study demonstrated that GLRA3 immunoreactivity is also present in the ventral horn (Wang et al., 2018). The latter study is more consistent with our observations as the *Glra3*-Cre(+) populations were localized in both the dorsal and ventral laminae (Fig. 1*A–C*), which is also in agreement with mRNA expression of *Glra3* (Ceder et al., 2023). To investigate the dendritic localization of *Glra3*-Cre(+) neurons, we performed cell fillings (Fig. 2*D*,*E*). Neurobiotin was used to fill the neurons and the morphology was revealed by staining the filled neuron with Alexa Fluor 488 streptavidin conjugate (Fig. 2*D*). Dendritic morphologies and locations are showed in Figure 2*E*. The dendritic tree of each filled neurons appeared to be local and without long projecting dendrites. All neurons showed vertical alignment, where the dendritic arbors projected predominantly in a dorsal–ventral direction.

### Adult *Glra3*-Cre(+) neurons are mainly located in laminae III–IV, and selective chemogenetic activation induces spontaneous behaviors indicative of a role in nociception and pruriception

**Figure 3.**
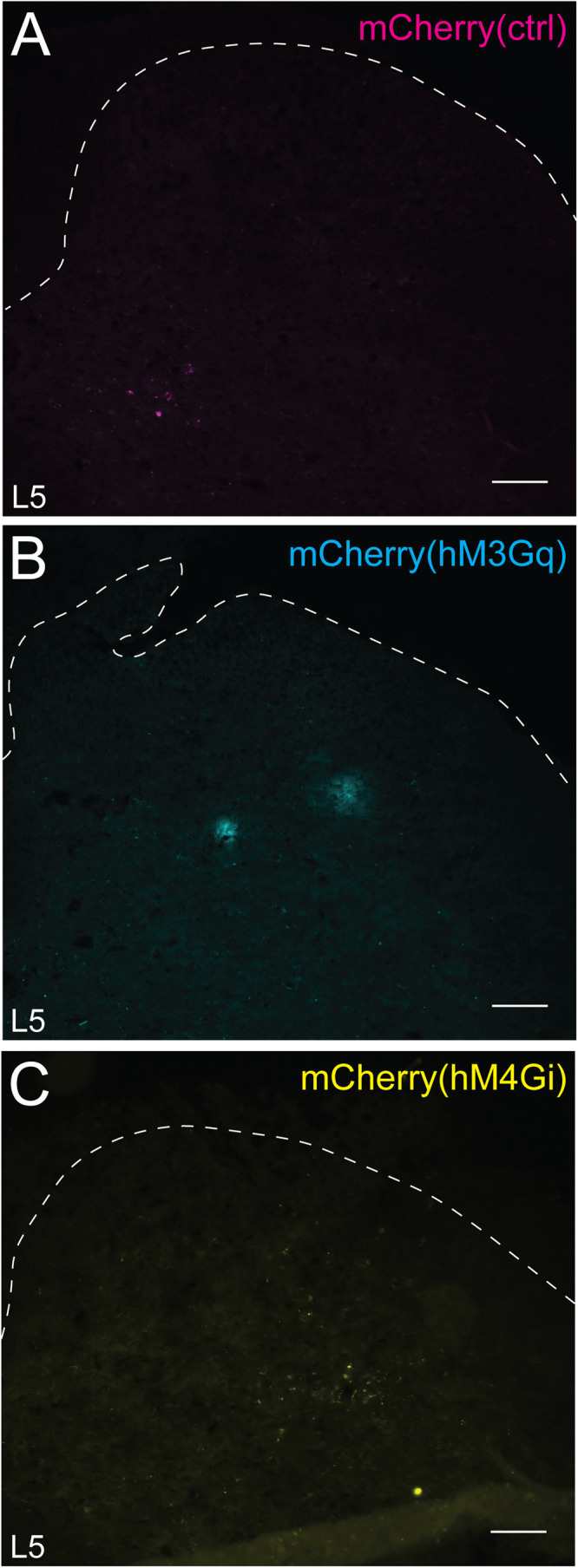
mCherry and chemogenetic viral vector fluorescent genes are not expressed in *Glra3*-Cre(−) wild-type injected mice. Low mCherry fluorescence detection, but no positive cells, could be visualized in close proximity to the L5/L6 injection site of AAV8.hSyn-DIO-mCherry (control virus, 2 females and 1 male; ***A***), AAV8.hSyn-DIO-hM3D(Gq)-mCherry (1 female and 2 males; ***B***), or AAV8.hSyn-DIO-hM4D(Gi)-mCherry (2 females and 1 male; ***C***) injected mice, demonstrating the Cre-dependent specificity of the viral vectors. Scale bar, 150 µm.

**Figure 4.**
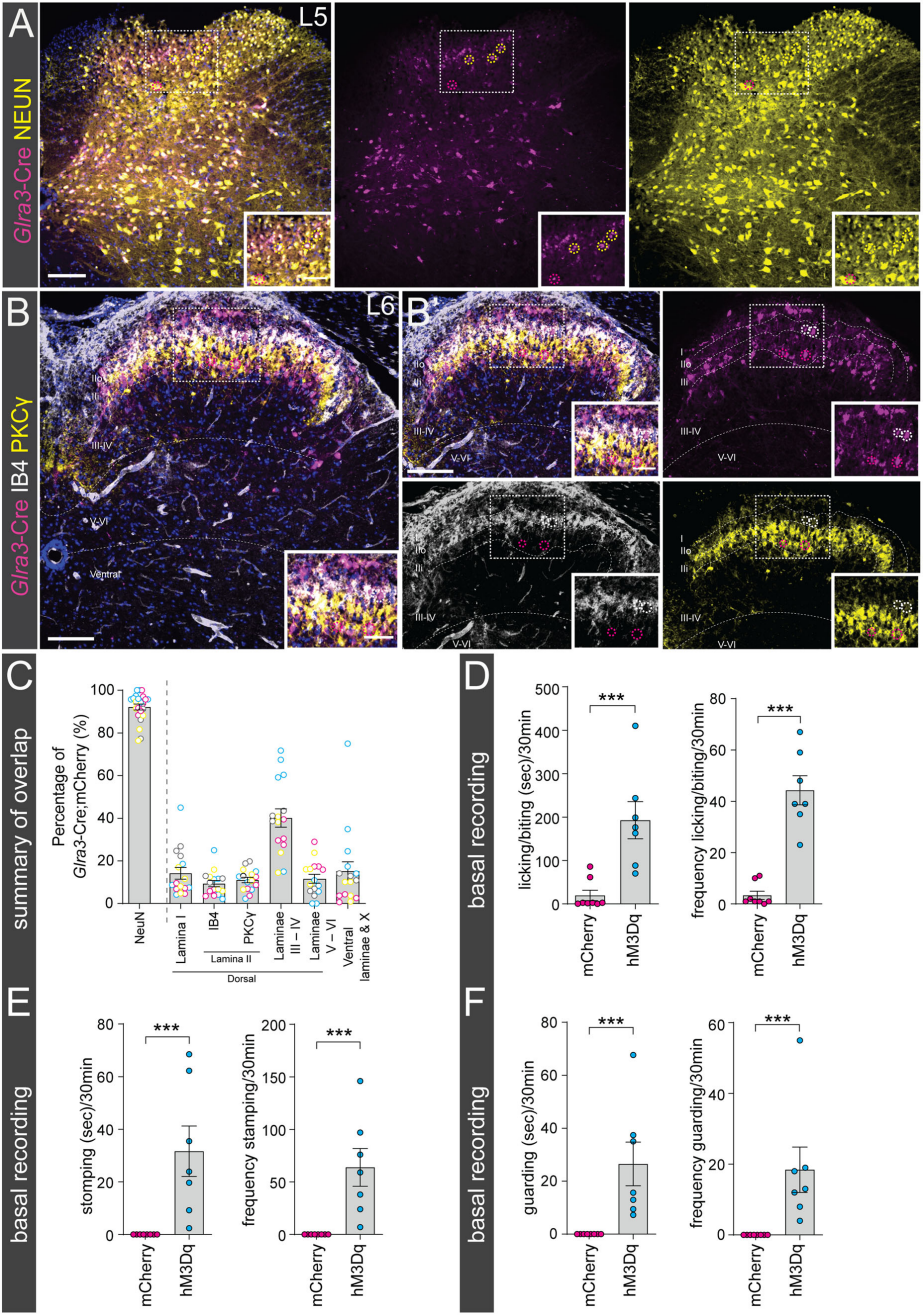
Adult *Glra3*-Cre(+) neurons are mainly located in laminae III–IV and selective chemogenetic activation induces spontaneous behaviors indicative of a role in nociception and pruriception. ***A***, Expression of NEUN (yellow) in *Glra3*-Cre.mCherry (magenta) lumbar spinal cord. Yellow dotted circles represent mCherry(+)NEUN(+) overlap and magenta dotted circles specify mCherry(+)NEUN(−) cells. ***B***, ***B’***, mCherry(+) colocalization with outer lamina II marker IB4 (white), inner lamina II marker PKCγ (yellow), and its expression in laminae III–IV, laminae V–VI, and ventral laminae defined from The Spinal Cord atlas (Anderson et al., 2009). The white dotted circles show mCherry(+) expression in the IB4 band and magenta dotted circles indicate mCherry(+)PKCγ(−) cells in the PKCγ band. mCherry(+)PKCγ(+) cells are not shown in this image. ***C***, Scatter bar plot of percentages of mCherry(+)NEUN(+) and laminae layer localization of mCherry(+) neurons (2 females, 2 males, *n* sections: NEUN: 31; PKCγ/IB4: 16). Scale bars: ***A***, ***B***, 100 µm; zoomed images, 50 µm. The observational dots in the scatter bar plots represent a unilateral part of the spinal cord, and different dot colors signify different mice. Results are presented as mean ± SEM. ***D–F***, Spontaneous behaviors, including licking/biting, stomping, and guarding of the corresponding dermatome (hindpaw/leg), were observed in *Glra3*-Cre.hM3Dq mice (7 + 8 mice; 7 females, 8 males) after 0.1 mg/kg intraperitoneal administration of CNO. ***D***, Chemogenetic activation of the *Glra3*-Cre populations increased total duration and frequency of licking/biting of the ipsilateral hindpaw compared with *Glra3*-Cre.mCherry mice in the 30 min time window post-CNO injection (duration, *p* = 0.0006; frequency, *p* = 0.0002). ***E***, Spontaneous stomping behavior was observed in *Glra3*-Cre.hM3Dq mice following CNO injection, which was not seen in *Glra3*-Cre.mCherry mice (duration and frequency, *p* < 0.0001). ***F***, *Glra3*-Cre.hM3Dq mice displayed guarding behaviors not observed in control mice. Both guarding duration and frequency were affected by *Glra3*-Cre population activation (*p* < 0.0001). Results are presented as mean ± SEM. Mann–Whitney *U* test was performed in ***D*** and chi-square test in ***E*** and ***F*** to compare the group means. **p < 0.001, ***p < 0.0001.

After the analysis of *Glra3*-Cre;*tdTomato* neurons, we further investigated the neuronal profile and anatomical location of adult *Glra3*-Cre(+) cells. Theoretically, the *tdTomato* reporter line marks both developmental and adult *Glra3*-Cre-expressing cells. Therefore, to label the adult population exclusively, reporter virus AAV8.hSyn-DIO-mCherry was unilaterally microinjected into the lumbar 5/lumbar 6 (L5/L6) spinal segments (abbreviated *Glra3*-Cre.mCherry). First, the specificity of the reporter, and the DREADD viral vectors used for the subsequent sensory behavioral analyses, were investigated by examining mCherry expression in *Glra3*-Cre(−) control mice. No fluorescent cells were detected (Fig. 3); thus the virally induced gene expression in subsequent analyses was Cre-dependent. The histological analysis (Fig. 4*A–C*) was conducted in the same manner as in the *Glra3*-Cre;*tdTomato* analysis, showing that 89.2 ± 3.9% (1,807/2,099) of the mCherry(+) cells coexpressed NEUN (Fig. 4*A*,*C*). In consistency with the tdTomato analysis, the mCherry(+) population was predominantly located in the dorsal horn (86.4 ± 3.2%, 1,279/1,524), with a minor subpopulation in the ventral horn (15.0 ± 4.5%, 245/1,524; Fig. 4*B*,*C*). In the dorsal horn, the mCherry(+) cells were mainly restricted to laminae III–IV (40.1 ± 4.2%, 590/1,524), followed by lamina I (14.2 ± 2.7%, 179/1,524), laminae V–VI (11.6 ± 2.0%, 222/1,524), the PKCγ(+) inner lamina II (11.1 ± 1.2%, 166/1,524) in which 21.3 ± 5.4% (41/166) of the mCherry(+) neurons were PKCγ(+), and the IB4(+) outer lamina II (9.4 ± 1.4%, 122/1,524; Fig. 4*B*,*C*).

Activation of spinal GLYT2 neurons decreases pain and itch behaviors (Foster et al., 2015), and the anatomical location of the *Glra3*-Cre(+) neurons showed herein indicates a sensory role of *Glra3*(+) neurons. To investigate this, *Glra3*-Cre(+) mice were unilaterally injected into L5/L6 with AAV8.hSyn-DIO-hM3D(Gq)-mCherry (abbreviated *Glra3*-Cre.hM3Dq) and the behavioral phenotype was compared with *Glra3*-Cre.mCherry mice (control; Fig. 3*D–F*). The mice were administered CNO to selectively activate the *Glra3*-Cre populations. After CNO administration, *Glra3*-Cre.hM3Dq mice displayed a higher duration and frequency of licking/biting of the ipsilateral hindpaw compared with control mice (Fig. 4*D*). In mice, licking of the hindpaw is associated with pain, while biting is a sign of itch (LaMotte et al., 2011). Therefore, our phenotype indicated both a nociceptive and a pruriceptive role for the *Glra3*-Cre populations. Additionally, activation of the *Glra3*-Cre.hM3Dq population resulted in stomping (Casarrubea et al., 2019) and guarding (Wang and Wang, 2003; Mogil and Crager, 2004), which were not observed in control mice (Fig. 4*E*,*F*). These behaviors further indicated nociceptive/pruriceptive-related roles of these populations. Collectively, activation of the lumbar spinal *Glra3*-Cre populations results in nocifensive and pruritofensive behaviors.

### Chemogenetic silencing of the *Glra3*-Cre populations decreases chloroquine- and compound 48/80-induced itch

**Figure 5.**
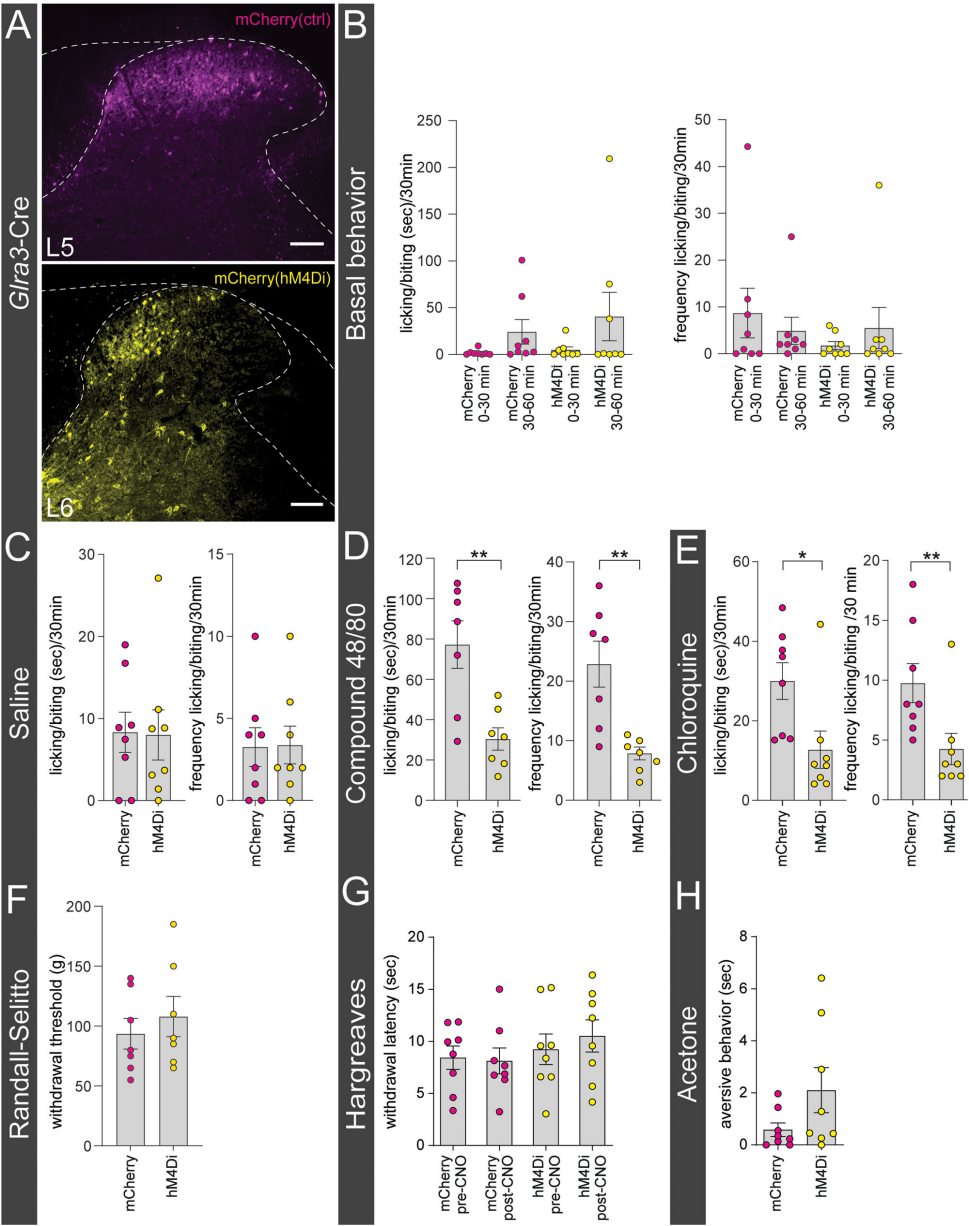
Chemogenetic silencing of *Glra3*-Cre(+) neurons decreases histaminergic and chloroquine-induced itch. ***A***, Dendritic and cytosolic expression of mCherry (magenta) after AAV8.hSyn-DIO-mCherry microinjection (top). Dendritic and cytosolic localization of hM4D(Gi)-mCherry (yellow) after microinjection of AAV8.hSyn-DIO-hM4D(Gi)-mCherry (bottom). ***B***, Intraperitoneal administration of CNO (0.1 mg/kg) did not induce spontaneous licking/biting of the affected dermatome in the 0–30 or 30–60 min intervals postinjection (duration: 0–30 min, *p* = 0.7463; 30–60 min, *p* = 0.4589; frequency: 0–30 min, *p* = 0.4109; 30–60 min, *p* = 0.3945, 8 mice/group; 11 females, 5 males). ***C***, Neither licking duration nor frequency were affected by saline administration (10 µl, duration, *p* = 0.7923; frequency, *p* = 0.9405, 8 mice/group; 9 females, 7 males). ***D***, Silencing *Glra3*-Cre(+) neurons attenuated the licking/biting duration and frequency following compound 48/80 (20 µg, 10 µl) injection (duration, *p* = 0.0037; frequency, *p* = 0.0028, 8 mice/group; 8 females, 8 males). ***E***, *Glra3*-Cre.hM4Di mice displayed lower licking/biting duration and frequency following chloroquine (10 mM, 10 µl) injection (duration, *p* = 0.0117; frequency, *p* = 0.0084, 8 mice/group; 9 females, 7 males). ***F***, Silencing of sacral *Glra3*-Cre(+) neurons did not affect the mechanical threshold in the tail (*p* = 0.5110, 7 mice/group, 7 females, 7 males). ***G***, ***H***, *Glra3*-Cre(+) neurons are not involved in thermal transmission. ***G***, Thermal stimulation (Hargreaves) of the ipsilateral hindpaw did neither affect the withdrawal latency in post-CNO administrated *Glra3*-Cre.hM4Di mice compared with *Glra3*-Cre.mCherry mice, nor the response pre- and postadministration of CNO in *Glra3*-Cre.hM4Di mice (*Glra3*-Cre.mCherry pre vs post CNO: *p* = 0.9981; *Glra3*-Cre.hM4Di pre vs post CNO: *p* = 0.9659; *Glra3*-Cre.mCherry vs *Glra3*-Cre.hM4Di post CNO: *p* = 0.5326, 8 mice/group; 11 females, 5 males). ***H***, Application of acetone solution (9:1 in water) did not affect the aversive response when silencing *Glra3*-Cre(+) neurons (*p* = 0.1145, 8 mice/group; 8 females, 8 males). Scale bar: ***A***, 100 µm. All results are presented as mean ± SEM. To compare the mean values, Mann–Whitney *U* test was performed in ***C*** (saline duration) and ***E***, unpaired two-tailed Student’s *t* test was performed in ***C*** (saline frequency), ***D***, ***F***, and ***H***. In ***G***, a one-way ANOVA with Šídák's multiple-comparisons test was used. *p < 0.05, **p < 0.01.

Since selective chemogenetic activation of *Glra3*-Cre(+) neurons induced spontaneous behaviors indicative of a role in pain/itch transmission, we sought to decipher the involvement of this population in different sensory modalities. For this purpose, adult *Glra3*-Cre(+) mice were unilaterally injected with AAV8-hSyn-DIO-hM4D(Gi)-mCherry in L5/L6 (abbreviated *Glra3*-Cre.hM4Di) to enable selective silencing while sensory behaviors were monitored. The results were compared with control virus-injected *Glra3*-Cre.mCherry mice (Fig. 5*A*). First, the basal behavioral phenotype was investigated following CNO administration. Selective silencing of *Glra3*-Cre(+) neurons did not affect spontaneous licking/biting behaviors in duration nor frequency during the 0–30 and 30–60 min intervals after CNO administration (Fig. 5*B*). Stomping and guarding behaviors were not observed when silencing the *Glra3*-Cre populations (data not shown).

In consistency with the Bourane et al. (2015) study, the mice were subjected to sensory testing 40 min after CNO administration. The pruriceptive role of the spinal lumbar *Glra3*-Cre population was examined in hairy skin, for which mice were administered either control saline, compound 48/80, or chloroquine solution (chemical itch) subcutaneously into the calf (Fig. 5*C–E*). Compound 48/80 activates sensory neurons both directly via MRGPRA1 (Schemann et al., 2012; Azimi et al., 2016, 2017) and indirectly as a mast cell degranulator by binding MRGPRB2 (Azimi et al., 2016), resulting in the release of pro-inflammatory molecules and pruritogens, including histamine and serotonin (Gupta and Harvima, 2018). Chloroquine activates primary afferents expressing MRGPRA3 (Liu et al., 2009). Saline evoked no differences in the duration or frequency of licking/biting of the injected area (Fig. 5*C*), showing that the *Glra3*-Cre populations do not convey sensory information associated with the injection itself. When administering compound 48/80, both the duration and frequency of licking/biting of the affected area were decreased following *Glra3*-Cre(+) silencing (Fig. 5*D*). For chloroquine administration, the same results were observed as with compound 48/80 injection (Fig. 5*E*).

The role of the *Glra3*-Cre populations in noxious mechanical transmission was examined using the Randall–Selitto test. To target the tail dermatome, AAV8-hSyn-DIO-hM4D(Gi)-mCherry or the control virus was injected in the sacral 2 (S2) segment (Bennett et al., 1999). The mechanical threshold for *Glra3*-Cre.hM4Di mice did not differ compared with *Glra3*-Cre.mCherry mice (Fig. 5*F*). To investigate if the *Glra3*-Cre populations are involved in thermal transmission, we performed the Hargreaves and acetone drop tests (Fig. 5*G*,*H*). Withdrawal response times, within groups, induced by heat stimulation of the ipsilateral hindpaw were not affected when comparing pre- and post-CNO administration in *Glra3*-Cre.mCherry or *Glra3*-Cre.hM4Di mice (Fig. 5*G*). When further comparing the withdrawal response times between the *Glra3*-Cre.mCherry and *Glra3*-Cre.hM4Di mice following CNO administration, no differences were observed (Fig. 5*G*). Application of a drop of acetone solution to the plantar surface of the ipsilateral hindpaw did not alter sensory responses, including flinching, withdrawal, or licking/biting of the paw (Fig. 5*H*). In conclusion, the *Glra3*-Cre populations have a pro-pruritic role in compound 48/80- and chloroquine-evoked itch, while not involved in acute noxious mechanical or thermal transmission.

### Spinal neurons activated by compound 48/80 or chloroquine coexpress *Glra3*

**Figure 6.**
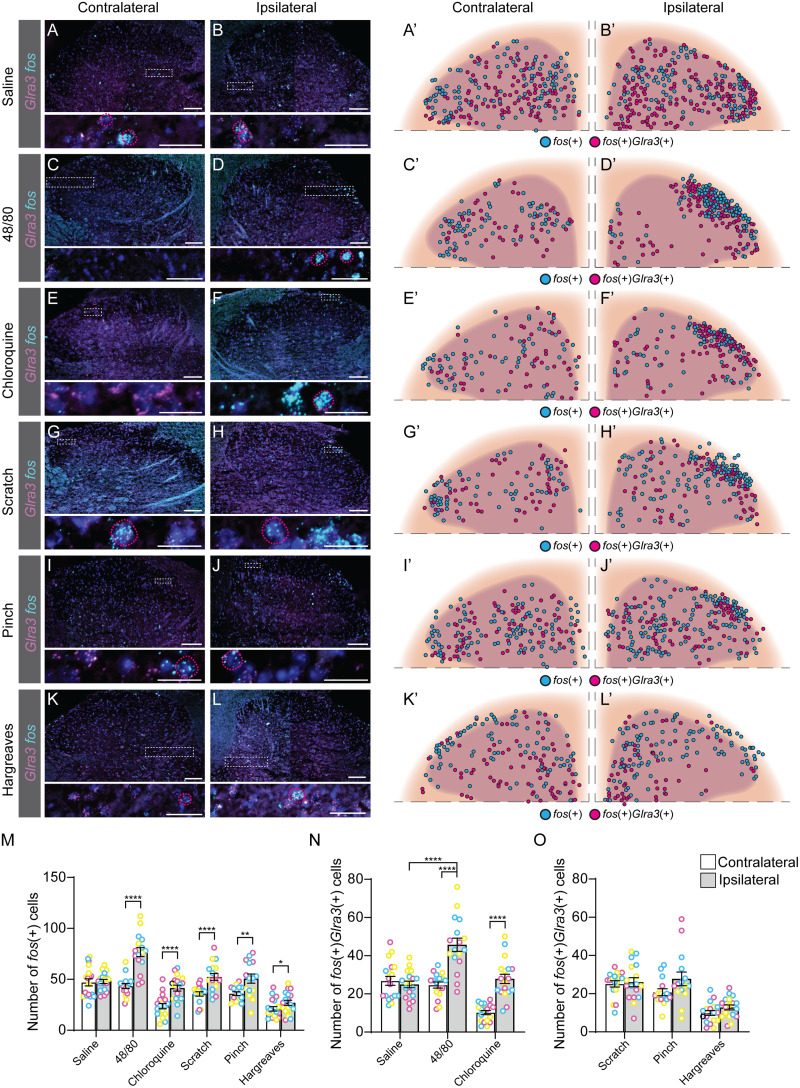
Spinal neurons activated by compound 48/80 or chloroquine coexpress *Glra3*. C57BL/6J mice were subjected to different sensory stimuli, whereafter the *Glra3* (magenta) and *fos* (cyan) coexpressional patterns were investigated (DAPI (dark blue)). ***A–B’***, Saline (10 µl; 1 female, 2 males); ***C–D’***, compound 48/80 (20 µg, 10 µl; 1 female, 2 males); ***E–F’***, chloroquine (20 mM, 10 µl; 1 female, 2 males); ***G–H’***, scratch [30 s, ∼300 mN (30.6 g); 2 females, 1 male]; and ***I–J’***, pinch (5 times for 5 s; 1 female, 2 males) in urethane-anesthetized mice (2 g/kg). ***K–L’***, Hargreaves (stimulated 3 times; 2 females, 1 male) in awake and freely moving mice. ***A–L***, Representative images of the contralateral and ipsilateral (stimulated side) dorsal horns for each stimulus, with close ups. *Fos*(+)*Glra3*(+) cells are depicted by a dotted magenta circle. Scale bars: ***A–L***, 100 µm; zoomed in ***A***, ***B***, ***E–J***, 20 µm; zoomed in ***C***, ***D***, ***K***, ***L***, 50 µm. To obtain high resolution, two images of each dorsal horn were acquired and later merged together, to a composited representative image of the dorsal horn, using Adobe Photoshop 22.3. ***A’–L’***, Schematic illustrations of the *fos*(+) and *fos*(+)*Glra3*(+)cells, where each cell is illustrated by a circle; *fos*(+) in cyan and *fos*(+)*Glra3*(+) in magenta (*n* sections; saline, 16; compound 48/80, 17; chloroquine, 17; artificial scratching, 16; pinch, 17; and Hargreaves, 18). ***M***, Scatter bar plot of the average number of *fos*(+) cells per dorsal horn for each stimulus on the contralateral (white bar) and ipsilateral (gray bar) side. ***N***, ***O***, Scatter bar plot of the average number of *fos*(+)*Glra3*(+) cells per dorsal horn for each stimulus on the contralateral (white bar) and ipsilateral (gray bar) side. Results are presented as mean ± SEM. Individual mice are marked with magenta, yellow, and cyan in ***M–O*** to display the spread between sections and mice. Paired two-tailed Student’s *t* tests were performed in ***M–O***, and a one-way ANOVA with Šídák's multiple-comparisons test to check for differences between saline, compound 48/80, and chloroquine injections. ***M***, Contralateral versus ipsilateral: saline, *p* = 0.8372; compound 48/80, *p* < 0.0001; chloroquine, *p* < 0.0001; artificial scratching, *p* < 0.0001; pinch, *p* = 0.0014; and noxious heat (Hargreaves), *p* = 0.0218. ***N***, The number of *fos*(+)*Glra3*(+) cells was higher on the ipsilateral side when injecting compound 48/80 or chloroquine compared with the contralateral side (saline, *p* = 0.5194; compound 48/80, *p* < 0.0001; and chloroquine, *p* < 0.0001). Compared with saline injections, only compound 48/80 injection resulted in a higher number of *fos*(+)*Glra3*(+) cells (*p* < 0.0001). ***O***, No differences in the number of *fos*(+)*Glra3*(+) neurons were detected for scratch (*p* = 0.6817), pinch (*p* = 0.0617), or Hargreaves (*p* = 0.1092). For separate channels, see Figure 7. For overlap with *fos*, *Glra3*, and *Vglut2* or *Viaat* for the following stimuli, scratching, pinch, and Hargreaves, see Figure 8.

**Figure 7.**
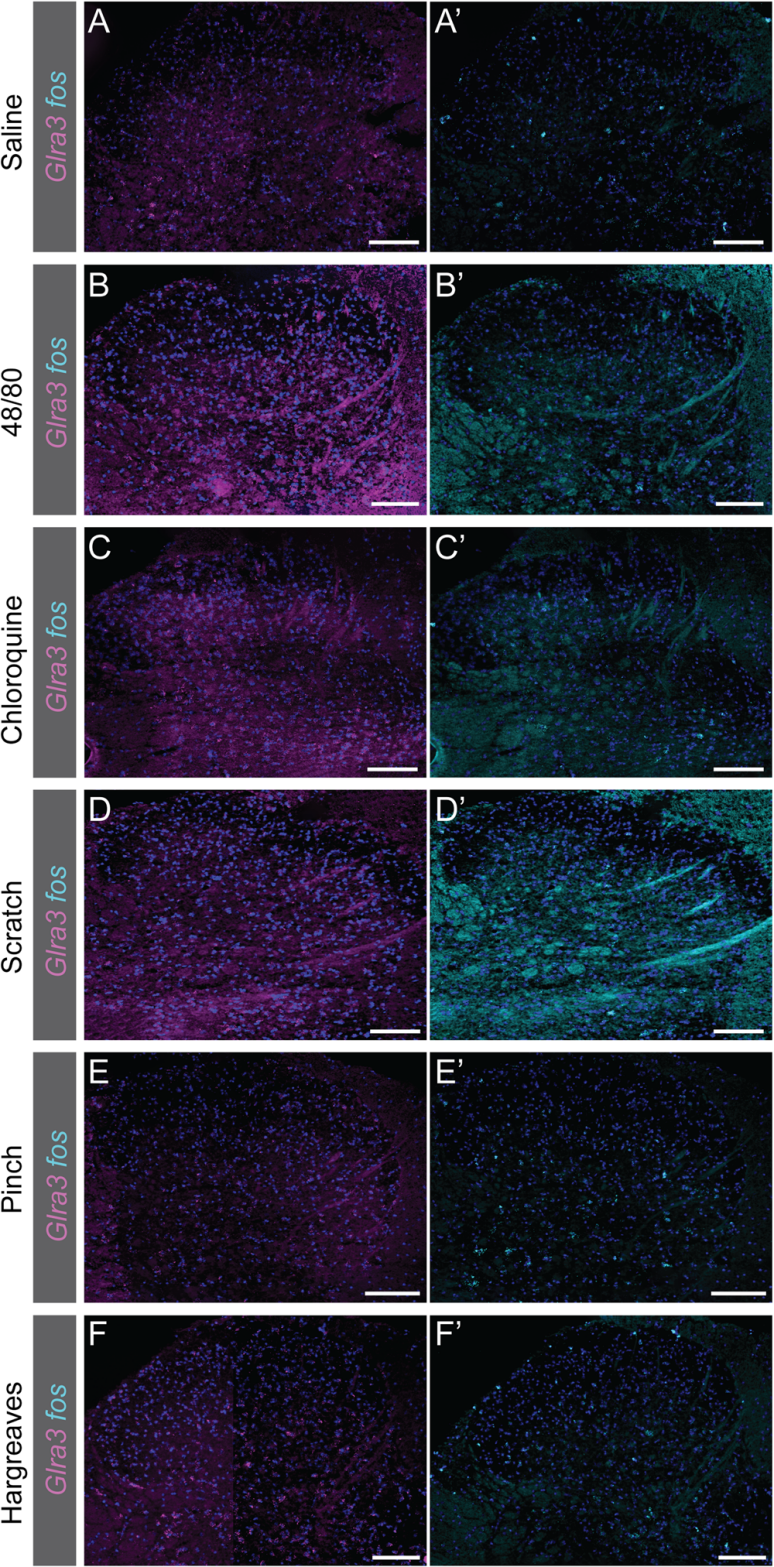
Expression of *Glra3* and *fos* in the contralateral L5/L6 dorsal horn following calf or paw stimulation in anesthetized or awake freely moving mice. ***A–F***, The expression of *Glra3* (magenta) in the contralateral L5/L6 dorsal horn following sensory stimulation. ***A’–F’***, Expression of *fos* (cyan) in the contralateral L5/L6 dorsal horn after sensory stimulation. ***A***, ***A’***, Saline (10 µl, 1 female, 2 males) injection in urethane (2 g/kg) anesthetized mice. ***B***, ***B’***, Compound 48/80 (20 µg, 10 µl, 1 female, 2 males) injection in urethane-anesthetized mice. ***C***, ***C’***, Chloroquine (20 mM, 10 µl, 1 female, 2 males) injection in urethane-anesthetized mice. ***D***, ***D’***, Scratching [30 s with 2 Hz and ∼300 mN (30.6 g), 2 females, 1 male] in urethane-anesthetized mice. ***E***, ***E’***, Pinching (5 times for 5 s, 1 female, 2 males) in urethane-anesthetized mice. ***F***, ***F’***, Hargreaves (stimulated 3 times, 2 females and 1 male) in freely moving awake mice. Scale bar: 50 µm. To obtain high resolution, two images of each dorsal horn were acquired and later merged together to a composited representative image of the dorsal horn using Adobe Photoshop 22.3.

**Figure 8.**
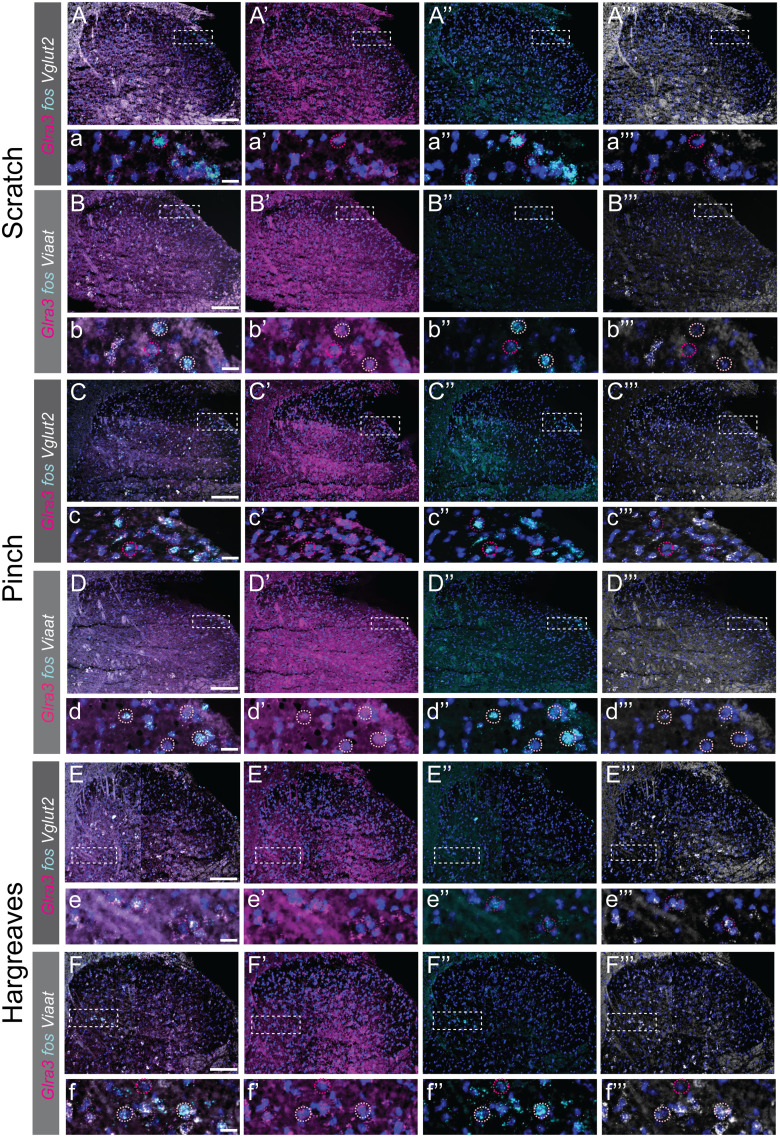
The expression of *Glra3*, sensory modality-induced *fos* cells, and *Vglut2* or *Viaat* in L5/L6 after dorsolateral calf or paw mechanical or heat stimulation in anesthetized or awake freely moving mice. ***A–E’’’***, Expressional view (***A–F***) of *Glra3* (’, magenta), *fos* (’’, cyan), and *Vglut2* or *Viaat* (’’’, white) in the L5/L6 ipsilateral dorsal horn after sensory stimulation of the dorsolateral calf in urethane (2 g/kg) anesthetized mice [scratch: 30 s with 2 Hz and ∼300 mN (30.6 g), 2 females, 1 male; pinch: 5 times for 5 s; 1 female, 2 males]. ***a–e’’’***, Zoomed in view of the respective marker genes after stimulation. ***F–F’’’***, Expressional view (***F***) of *Glra3* (***F’***, magenta), *fos* (***F’’***, cyan), and *Vglut2* or *Viaat* (***F’’’***, white) in the L5/L6 ipsilateral dorsal horn after noxious heat stimulation of the hindpaw (stimulated 3 times with 20 s cutoff time; 2 females, 1 male) in awake freely moving mice. ***f–f’’’***, Zoomed in view of the respective marker genes after noxious heat stimulation. Magenta dotted circles show *fos*(+)*Glra3*(+), light pink indicates *fos*(+)*Viaat*(+), and dark magenta shows *fos*(+)*Glra3*(+)*Vglut2*(+). Scale bars: ***A–F***, 50 µm; ***a–f’’’***, 20 µm. To obtain high resolution, two images of each dorsal horn were acquired and later merged together to a composited representative image of the dorsal horn using Adobe Photoshop 22.3.

**Figure 9.**
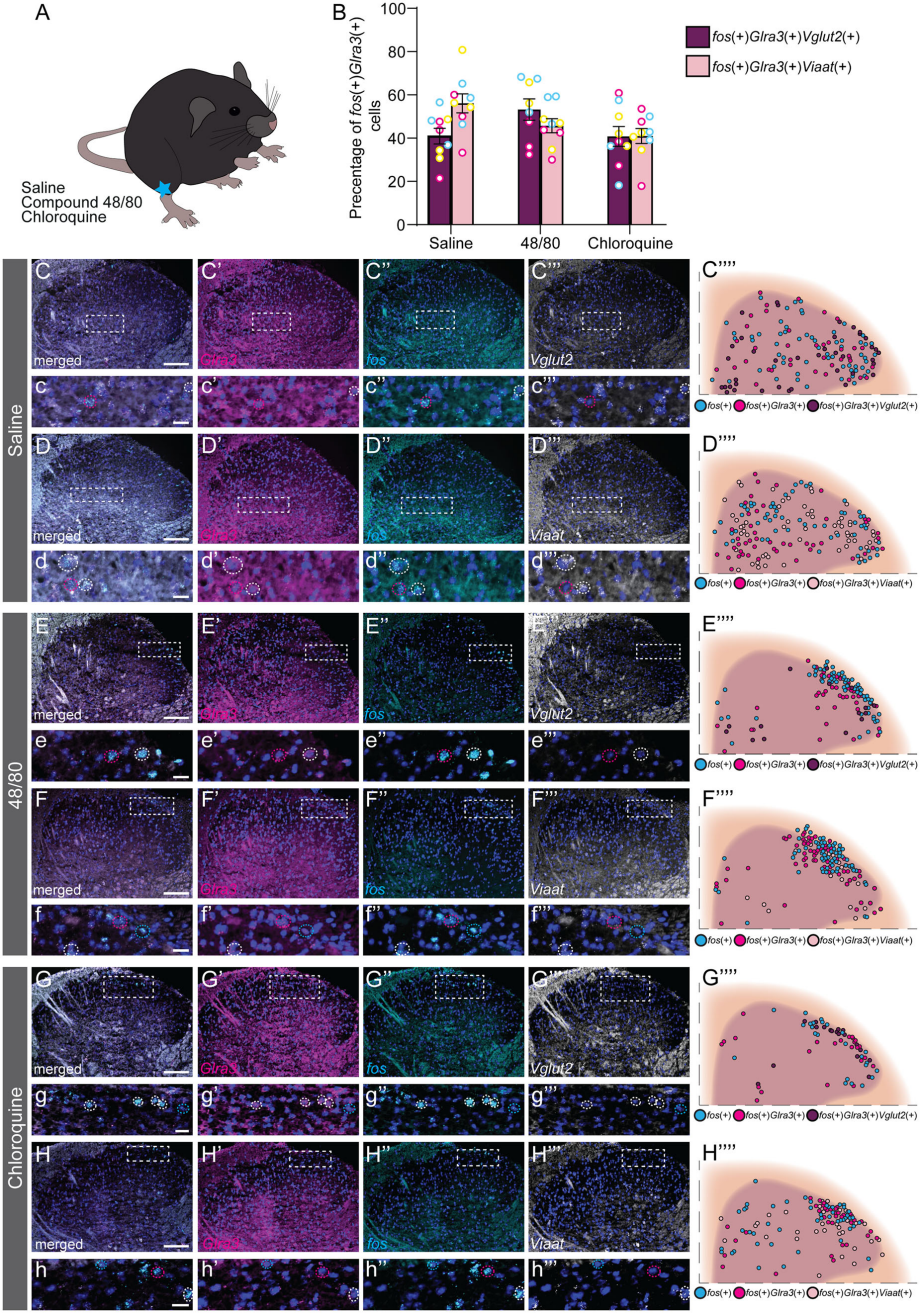
Compound 48/80- and chloroquine-induced *fos* cells expressing *Glra3* are both excitatory and inhibitory. Expression of *fos*, *Glra3*, and *Vglut2* or *Viaat*, and the ipsilateral L5/L6 dorsal horn spatial location of cells expressing these genes (3 mice of mixed sex/stimulation, *n* sections/mice: 2–4), following control saline (10 µl, *n* sections: 9), compound 48/80 (20 µg, 10 µl, *n* sections: *Vglut2*: 8; *Viaat*: 9), or chloroquine (20 mM, 10 µl, *n* sections: *Vglut2*: 9; *Viaat*: 9) calf injections in urethane (2 g/kg) anesthetized mice. ***A***, Schematic illustration of calf injections. ***B***, The coexpression of *Vglut2* or *Viaat* in saline, compound 48/80-, and chloroquine-activated *fos*(+)*Glra3*(+) cells. Both excitatory *Glra3*(+)*Vglut2*(+) and inhibitory *Glra3*(+)*Viaat*(+) neurons are activated following saline, compound 48/80, and chloroquine administration. Saline-activated *fos*(+)*Glra3*(+) population showed 40.9 ± 3.6% (94/231) coexpression with *Vglut2* and 56.1 ± 4.4% (124/218) with *Viaat*; compound 48/80 displayed 53.2 ± 4.9% (208/373) and 45.7 ± 3.2% (196/422) coexpression with *Vglut2* and *Viaat*, respectively. Chloroquine-activated *fos*(+)*Glra3*(+) neurons coexpressed both *Vglut2* and *Viaat* in similar proportions: 40.8 ± 4.6% (98/238) and 40.9 ± 3.5% (107/264), respectively. The graph presents data as mean ± SEM. ***C–H’’’’***, Each panel shows the overview of the expression of the targeted genes with nucleus marker DAPI (dark blue) first, followed by *Glra3* (’, magenta), sensory stimulation-induced *fos* (’’, cyan), and *Vglut2* or *Viaat* (’’’, white). Magenta dotted circle: *fos*(+)*Glra3*(+) cells; white dotted circle: *fos*(+)*Glra3*(+)*Vglut2*(+) or *fos*(+)*Glra3*(+)*Viaat*(+) cells. The schematic images in (’’’’) show the spatial localization of the sensory-induced *fos*(+) cells (cyan), *fos*(+)*Glra3*(+) (magenta), and *fos*(+)*Glra3*(+)*Vglut2*(+) (***C’’’’***, ***E’’’’***,***G’’’’***; purple) or *fos*(+)*Glra3*(+)*Viaat*(+) (***D’’’’***, ***F’’’’***, ***H’’’’***; light pink) cells. ***C’’’’***, ***D’’’’***, Saline injection resulted in a widespread *fos*(+) cell pattern in the dorsal horn, and overlapping cells with *Glra3*(+) could be found in the whole dorsal horn. Moreover, the saline-activated *fos*(+)*Glra3*(+)*Vglut2*(+) neurons were found to be located more to the lateral part of the dorsal horn, while the saline-activated *fos*(+)*Glra3*(+)*Viaat*(+) cells were more spread over the dorsal horn, with some clustering in the medial part of the dorsal horn. ***E’’’’***, ***F’’’’***, Compound 48/80-activated *fos*(+) cells were clustered in the superficial layer of the dorsolateral horn, where *Glra3*(+)*Vglut2*(+) cells (***E’’’’***) and *Glra3*(+)*Viaat*(+) cells (***F’’’’***) were found in the same area. ***G’’’’***, ***H’’’’***, Chloroquine-activated *fos*(+) cells clustered in similar patterns as observed for compound 48/80, but the chloroquine *fos*(+) cells were fewer in number compared with compound 48/80. *Fos*(+)*Glra3*(+)*Vglut2*(+) cells (***G’’’’***) were found more dorsolateral, similar to *fos*(+)*Glra3*(+)*Viaat*(+) cells (***H’’’’***), which were found mostly dorsolateral but with a higher degree of scattering. Scale bars: ***C–H***, 100 µm; ***c–h***, 50 µm. To obtain high resolution, two images of each dorsal horn were acquired and later merged together to a composited representative image of the dorsal horn using Adobe Photoshop 22.3.

Based on the behavioral observations, we wanted to molecularly verify the proposed sensory role of spinal *Glra3*(+) neurons and subsequently relate it to the *Vglut2*(+) and *Viaat*(+) spinal *Glra3*-Cre subpopulations. To do so, sensory stimulations in anesthetized and awake C57BL/6J mice were performed, followed by RNAscope (Wang et al., 2012) analyses of *fos* (Sheng and Greenberg, 1990), *Glra3*, and *Vglut2* or *Viaat* in the L4/L6 dorsal spinal cord. The mice were subjected to one of six possible stimuli: a subcutaneous injection of saline, compound 48/80, or chloroquine subcutaneously in the right dorsolateral calf, a noxious mechanical stimulus (pinch or scratching) of the right dorsolateral calf, or thermal (Hargreaves) stimulation of the right hindpaw (Fig. 6*A–L’*; for separate channels see Figs. 7, 8). Scratching of the calf is not a natural behavior of mice; however, this stimulation was conducted in this area to enable comparison with the other stimuli. To prevent transcriptional influence from pain- and itch-responsive behaviors, we performed all stimulations under urethane anesthesia, except for the Hargreaves test that was performed on awake freely moving mice. All stimuli, except saline, were found to have a higher number of *fos*(+) cells on the ipsilateral side compared with the contralateral side: saline contralateral 47 ± 5 (847) and ipsilateral 48 ± 2 (862); compound 48/80 contralateral 43 ± 3 (779) and ipsilateral 75 ± 5 (1,347); chloroquine contralateral 24 ± 5 (426) and ipsilateral 41 ± 3 (745); artificial scratching contralateral 36 ± 3 (606) and ipsilateral 52 ± 4 (889); pinch contralateral 36 ± 3 (573) and ipsilateral 52 ± 12 (834); and noxious heat (Hargreaves) contralateral 21 ± 3 (378) and ipsilateral 27 ± 2 (485) (Fig. 6*M*). *Fos*(+)*Glra3*(+)-expressing cells were found to be greater in number on the ipsilateral dorsal horn than those on the contralateral dorsal horn for both compound 48/80 and chloroquine (Fig. 6*N*). The average number of *fos*(+)*Glra3*(+) cells in the ipsilateral dorsal horn after injection with compound 48/80 was higher than the average number of *fos*(+)*Glra3*(+) cells after saline injection, which was not observed for chloroquine administration (Fig. 6*N*). Of *fos*(+) cells, more than half of compound 48/80- and chloroquine-activated cells expressed *Glra3* [compound 48/80, 59% (795/1,347); chloroquine, 67% (502/745); saline, 52% (449/862); scratch, 50% (442/889); pinch, 49% (218/443); Hargreaves, 47% (230/486)]. No difference in the number of *fos*(+)*Glra3*(+) expressing cells could be detected for scratch, pinch, or Hargreaves (comparing the ipsi- and contralateral sides; Fig. 6*O*). Altogether, these findings verify that *Glra3*(+) neurons are involved in the communication of compound 48/80- and chloroquine-induced itch and that these neurons are not involved in acute mechanical or thermal transmission. Since *Glra3* is found in both excitatory and inhibitory neuronal populations (Häring et al., 2018; Zeisel et al., 2018), we further investigated the sensory modality activation of these *fos*(+)*Glra3*(+) subpopulations after injections with saline, compound 48/80, or chloroquine, focusing on the ipsilateral dorsal horn (Fig. 9*A–H’’’’*). All three stimuli led to the expression of *fos* in both *Glra3*(+)*Vglut2*(+) and *Glra3*(+)*Viaat*(+) populations (Fig. 9*B*). Taken together, the transcriptional analysis shows that *Glra3* is expressed in compound 48/80- and chloroquine-activated neurons, suggesting a role in transmission of these two sensory stimuli. Moreover, the sensory modality activated *fos*(+)*Glra3*(+) cells can be found in subpopulations expressing the excitatory marker *Vglut2*(+) or the inhibitory marker *Viaat*(+).

### Lumbar *Glra3*-Cre(+) neurons receive monosynaptic input from excitatory and inhibitory local spinal neurons

**Figure 10.**
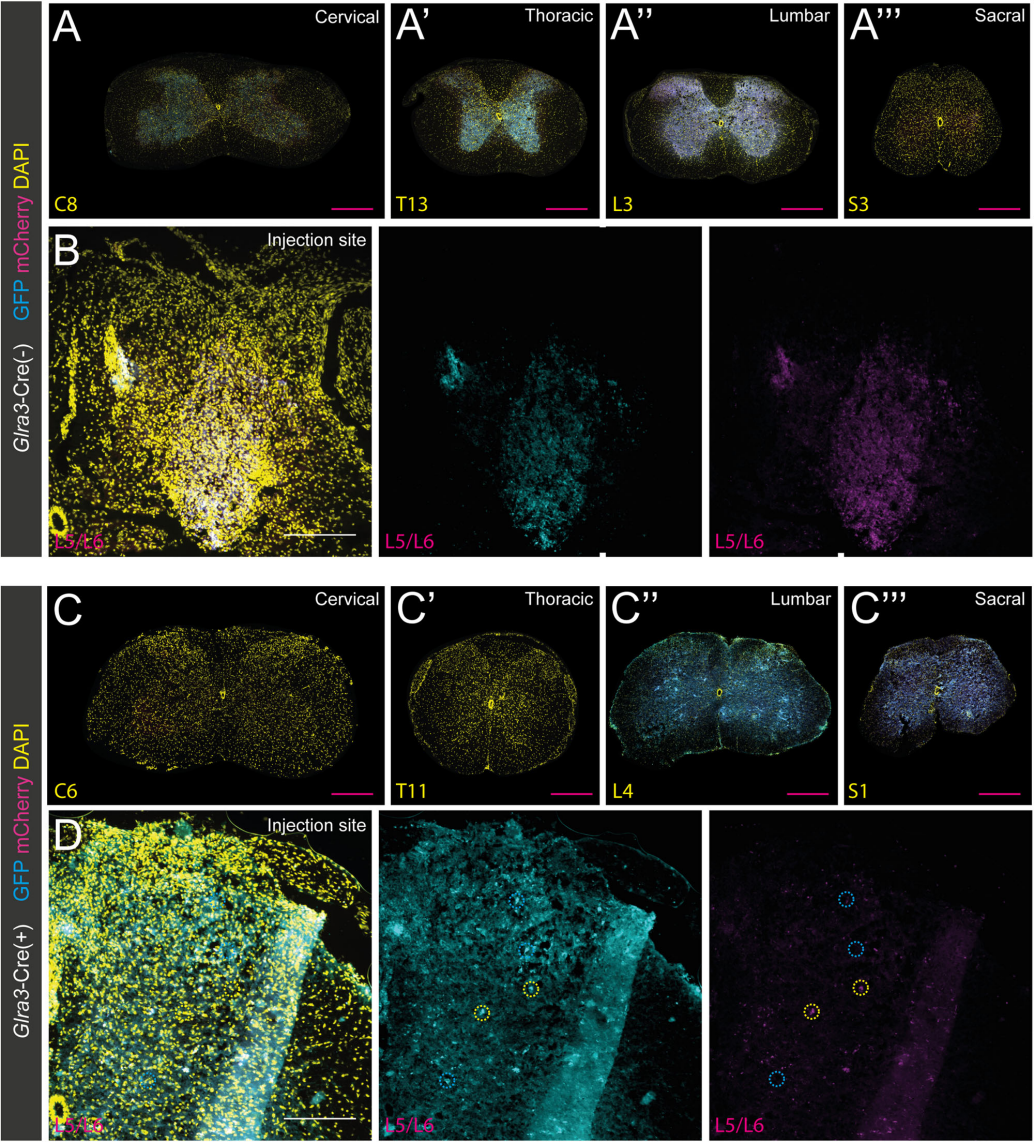
Starter and traced cells were mainly found in the lumbar division in *Glra3*-Cre(+) mice and only a few cells were detected in *Glra3*-Cre(−) mice. ***A–A’’’***, No starter GFP(+)mCherry(+) or traced mCherry(+) cells were detected in the thoracic (***A’***), lumbar (***A’’***), or sacral (***A’’’***) divisions in *Glra3*-Cre(−) mice. Two mCherry(+) cells were found in the cervical (***A***) division in two separate mice, where 1 mCherry(+) cell was found in the contralateral ventral horn and 1 cell in the ipsilateral dorsal horn (3 females, 3 males, every sixth section analyzed). These cells are not depicted in the image. ***B***, No starter GFP(+)mCherry(+) or traced mCherry(+) cells were observed at the injection site of *Glra3*-Cre(−) mice. ***C***, Starter GFP(+)mCherry(+) and traced mCherry(+) cells were found in the lumbar division (***C’’***) and 1 mCherry(+) cell was observed in the distant cervical (***C***), whereas no traced cells were located in the thoracic (***C’***) or sacral (***C’’’***) divisions in *Glra3*-Cre(+) mice (5 females, 5 males, every sixth section analyzed). ***D***, Starter GFP(+)mCherry(+) and traced mCherry(+) cells were detected at the injection site of *Glra3*-Cre(+) mice. The blue dotted circles represents GFP(+)mCherry(−) cells and the yellow dotted circles show GFP(+)mCherry(+) starter cells. Traced mCherry(+) is not displayed in images. GFP is displayed as cyan and mCherry as magenta (DAPI as yellow). Scale bars: ***A–A’’’***, ***C–C’’’***, 300 µm; ***B***, ***D***, 150 µm. For high resolution, images were acquired in 10× and were thereafter merged for representation in Adobe Photoshop 22.3.

After identifying a pro-pruritic role for *Glra3*-Cre(+) neurons via behavioral experiments and coexpression of *Glra3* in compound 48/80- and chloroquine-activated *fos*(+) cells, we investigated the connectivity of lumbar *Glra3*-Cre(+) neurons. Retrograde viral tracing and dorsal root stimulation were used to deduce the mono- and polysynaptic neurons targeting the *Glra3*-Cre populations. To enable analysis of the monosynaptic connectivity, we performed a two-step viral injection procedure. First, the helper virus AAV8.Syn-flex-TVA-oG-GFP was injected, enabling *Glra3*-Cre(+) host cell entry and subsequent retrograde monosynaptic propagation of the secondly injected EnvA pseudotyped mCherry rabies virus. In the spinal cord of control *Glra3*-Cre(−) mice, no helper GFP(+)mCherry(−) nor starter GFP(+)mCherry(+) cells were detected. Two mCherry(+) cells were found in the cervical division [1 cell in the ipsilateral dorsal horn and 1 mCherry(+) cell in the contralateral ventral horn (one in each mouse); Fig. 10*A*,*B*]. In the brain, no traced mCherry(+) cells were detected in control mice. In the lumbar DRG, 51 mCherry(+) cells (43 ipsilateral, 8 contralateral) were found in two mice (43 ipsilateral and 6 contralateral in one mouse and 2 contralateral in a second mouse), verifying the Cre-dependent robustness and reliability of this tracing system.

In the spinal cords of *Glra3*-Cre(+) mice, 94 starter GFP(+)mCherry(+) cells were localized in the lumbar enlargement (Fig. 10*C’’*,*D*). Furthermore, 526 traced mCherry(+) cells were found in the ipsilateral lumbar enlargement, and in four out of five mice, 16 traced cells were found in the contralateral lumbar spinal cord. None of these mice had any starter GFP(+)mCherry(+) cells located on the contralateral side (Fig. 10*C*,*D*). Thus, it is possible that the *Glra3*-Cre populations receive some input from the contralateral side in addition to abundant ipsilateral input. Also, one mCherry(+) cell was detected in the ipsilateral dorsal horn of the cervical division, while none were detected in either thoracic or sacral divisions (Fig. 10*C–C’’’*), indicating that the *Glra3*-Cre populations receive mainly local spinal input.

**Figure 11.**
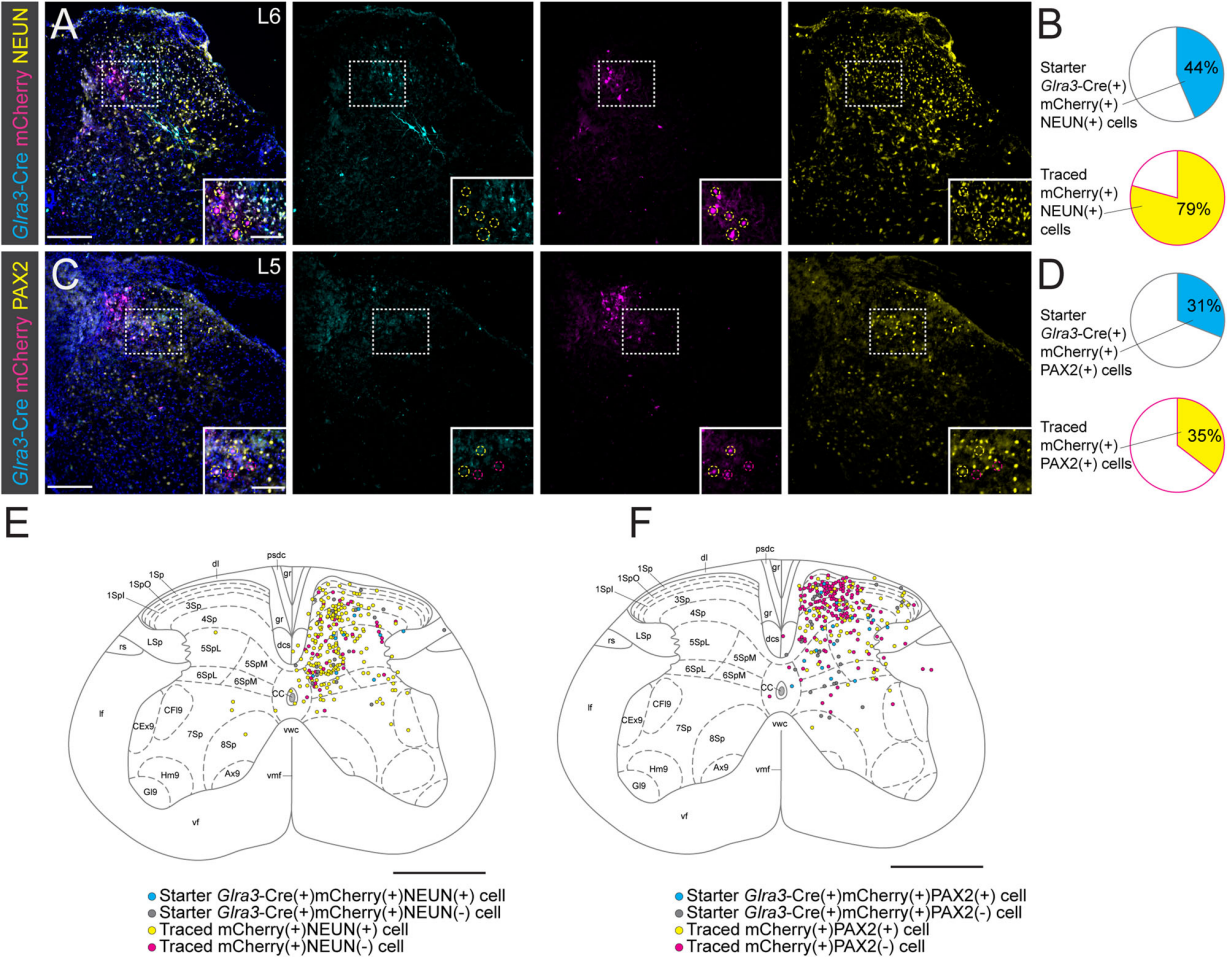
Lumbar *Glra3*-Cre(+) neurons receive monosynaptic input from excitatory and inhibitory local spinal neurons. ***A***, Coexpression of NEUN (yellow) in *Glra3*-Cre starter GFP(+)mCherry(+) and traced mCherry(+) cells. ***B***, Pie charts of NEUN overlap in ipsilateral starter *Glra3*-Cre(+) (top chart) and traced cells (bottom chart; 3 females, 2 males, *n* images: 29). ***C***, Colocalization of PAX2 (yellow) in *Glra3*-Cre starter GFP(+)mCherry(+) and traced mCherry(+) cells. GFP is displayed as cyan and mCherry as magenta. ***D***, Pie charts of coexpression of PAX2 in the starter *Glra3*-Cre(+) (top chart) and traced cells (bottom chart; 3 females, 2 males, *n* images: 31). Scale bars: ***A***,***C***, 150 µm; zoomed images, 75 µm. ***E***, Schematic illustration of the spatial localization of NEUN(+) and NEUN(−) *Glra3*-Cre starter GFP(+)mCherry(+) and traced mCherry(+) cells in the ipsi- and contralateral spinal lumbar division. ***F***, Schematic illustration of *Glra3*-Cre starter GFP(+)mCherry(+) and traced mCherry(+) cell localizations and overlap with PAX2 in the ipsi- and contralateral spinal lumbar division. The marker(+) starter cells are shown as cyan dots and the marker(−) starter cells as gray dots, whereas the marker(+) traced cells are depicted as yellow dots and marker(−) traced cells as magenta dots. The schematic image was acquired from Atlas of the Mouse Spinal Cord (Watson and Paxinos, 2009). For starter and traced cells in *Glra3*-Cre(−) mice and the cervical, thoracic and sacral divisions of the spinal cord in *Glra3*-Cre(+) mice, please see Figure 10.

To molecularly examine the starter and traced cells, we investigated the colocalizations with NEUN and the inhibitory marker PAX2 (Larsson, 2017). Starter cells overlapped 44% (17/39), whereas 79% (191/241) of the traced cells colocalized with NEUN (Fig. 11*A*,*B*). 30.9% (17/55) of the starter cells, and 35.4% (101/285) of the traced cells overlapped with PAX2 (Fig. 11*C*,*D*), which, in consistency with the RNAscope findings, further indicates that the *Glra3*-Cre population comprises an inhibitory subpopulation. In the dorsal–ventral axis, the mCherry(+) cells were mainly located in the dorsal horn (laminae I–VI; Fig. 11*E*,*F*), suggesting that the spinal input to the *Glra3*-Cre populations predominately constitutes of sensory-related transmission. A smaller subpopulation of mCherry(+) cells was observed in the ventral horn (laminae VII–IX) and lamina X (Fig. 11*E*,*F*), with the former suggesting that the *Glra3*-Cre populations potentially receive input from motor-related spinal neurons.

### Lumbar *Glra3*-Cre(+) neurons receive monosynaptic input from several brain areas

**Figure 12.**
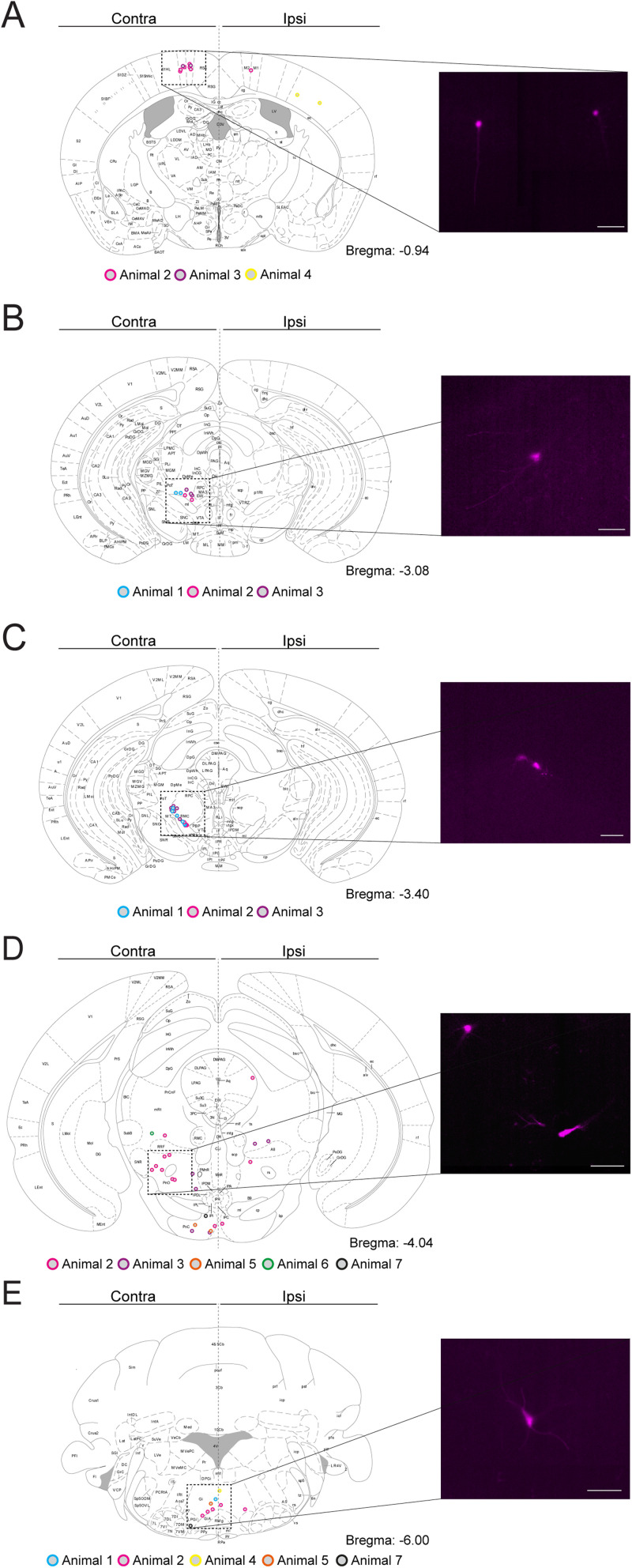
Lumbar *Glra3*-Cre(+) neurons receive monosynaptic input from several brain areas. ***A–D***, Schematic illustrations of the mono- and presynaptic traced mCherry(+) cells found in several brain areas [*n* cells = 89 from 7 out of the 10 mice; 5 females, 5 males; 3 females and 4 males had traced mCherry(+) cells]. The brain areas with more than one mCherry(+) cell or the brain areas with mCherry(+) cells in more than one mouse are shown in the figure. The coloring of the dots represents the different mice and the schematic images were acquired from The Mouse Brain Atlas *in Stereotaxic Coordinates* (Paxinos and Franklin, 2001). ***A***, Traced mCherry(+) cells in the ipsilateral and contralateral primary and secondary motor cortices (M1, M2; bregma: −0.94–(−)1.22 mm; 2 females) and in the ipsilateral somatosensory cortex, barrel field [S1BF; bregma: 0.38–(−)1.34 mm; 1 male]. ***B***, mCherry(+) cells were observed in the contralateral p1 reticular formation [p1Rt; bregma:−3.08–(−)3.16 mm; 3 females]. ***C***, The contralateral localization of mCherry(+) cells in the magnocellular and parvicellular parts [RMC and RPC; bregma: −3.08–(−)4.04 mm; 3 females]. ***D***, The ipsilateral and contralateral localization of traced mCherry(+) cells in the oral and caudal part of the pontine reticular nucleus [PnO and PnC; bregma: −4.24–(−)5.23 mm; 2 females, 2 males]. ***E***, mCherry(+) cells were bilaterally localized in the gigantocellular vestibular nucleus [Gi; bregma: −5.88–(−)6.97 mm; 2 females, 2 males]. Scale bar, 100 µm.

In the brain, a total of 89 traced mCherry(+) cells were detected in seven out of 10 *Glra3*-Cre(+) mice. One mouse had a traced cell in the ipsilateral and two mice had traced cells in the contralateral motor cortices (M1, M2; *n* cell = 9). In a third mouse, traced cells were located in the ipsilateral somatosensory cortex, barrel field (S1BF; *n* cells = 2) area (Fig. 12*A*). Three mice had mCherry(+) cells in the contralateral p1 reticular formation (p1Rt; *n* cells = 7; Fig. 12*B*) and in the red nucleus magnocellular part/red nucleus parvicellular part (RPC/RMC; *n* cells = 16 cells; Fig. 12*C*). In addition, traced cells were detected in the ipsilateral and contralateral pontine reticular nucleus, either in the oral (PnO; *n* cells = 10) or caudal part (PnC; *n* cells = 7; Fig. 12*D*), and bilaterally in the gigantocellular vestibular nucleus (Gi; *n* cells = 8; Fig. 12*E*). This demonstrates that the lumbar *Glra3*-Cre populations receive monosynaptic input from several brain areas. For details regarding brain area localization of the traced mCherry(+) cells in the individual mice, see Table 3.

**Table 3.** Brain areas containing spinal lumbar *Glra3*-Cre(+) retrogradely traced neurons

Sex	Animal ID	Bregma	Brain structure abbreviation	Brain structure	Number of cells	Ipsi/Contra
Female	1	−3.08–(−4.04)	RMC	Red nucleus, magnocellular part	5	Contra
		−3.08	p1Rt/REth	p1 reticular formation/retroethmoid nucleus	1	Contra
		−3.16	p1Rt	p1 reticular formation	1	Contra
		−3.52	RMC/RPC	Red nucleus, magnocellular part/red nucleus, parvicellular part	1	Contra
		−3.80	PaR	Paratubal nucleus	1	Contra
		−5.80	IRt	Intermediate reticular nucleus	2	Contra
		−5.88	MVeMC	Medial vestibular nucleus, magnocellular part	1	Ipsi
		−5.88	MVePC	Medial vestibular nucleus, parvicellular part	2	Contra
		−6.12	Gi	Gigantocellular vestibular nucleus	1	Contra
		−6.96	MVe	Medial vestibular nucleus	1	Contra
Female	2	−0.94	M1	Primary motor cortex	3	Contra
		−0.94	M2	Secondary motor cortex	3	Contra
		−1.22	M1/M2	Primary motor cortex/secondary motor cortex	1	Ipsi
		−3.08	p1Rt	p1 reticular formation	2	Contra
		−3.16	RMC/RPC	Red nucleus, magnocellular part/red nucleus, parvicellular part	2	Contra
		−3.40	RMC	Red nucleus, magnocellular part	2	Contra
		−4.04	LPAG	Lateral periaqueductal gray	1	ipsi
		−4.04	Su3	Supraoculomotor periaqueductal gray	1	Ipsi
		−4.24	mRT	Mesencephalic reticular formation	1	Contra
		−4.24	PTg/PnO	Reticulotegmental nucleus of the pons/pontine reticular nucleus, oral part	1	Contra
		−4.48–(−4.60)	PnO	Pontine reticular nucleus, oral part	7	1 ipsi, 6 contra
		−5.20	PnC	Pontine reticular nucleus, caudal part	2	1 ipsi, 1 contra
		−5.20	PO	Paraolivary nucleus	1	Contra
		−5.68	SuVe	Superior vestibular nucleus	1	Contra
		−5.88	SuVe/LVe	Superior vestibular nucleus/lateral vestibular nucleus	1	Contra
		−5.88–(−7.08)	Gi	Gigantocellular vestibular nucleus	5	2 ipsi, 3 contra
		−6.24–(−7.08)	SpVe	spinal vestibular nucleus	3	1 ipsi, 2 contra
		−6.96	C1	C1 adrenaline cells	1	Ipsi
		−7.08	Sol	Solitary tract	1	Ipsi
Female	3	−1.22	M1	Primary motor cortex	1	Contra
		−1.22	M2	Secondary motor cortex	1	Contra
		−3.08	p1Rt	p1 reticular formation	3	Contra
		−3.28	mRt	Mesencephalic reticular formation	2	Ipsi
		−3.28	RMC	Red nucleus, magnocellular part	1	Contra
		−3.28	RPC	Red nucleus, parvicellular part	5	Contra
		−4.24–(−4.60)	PnO	Pontine reticular nucleus, oral part	2	Contra
		−4.60	Pa4	Paratrochlear nucleus	1	
		−5.20	PnC	Pontine reticular nucleus, caudal part	2	Contra
		−5.20	PnR	Pontine raphe nucleus	1	Ipsi
		−5.88	MVeMC	Medial vestibular nucleus, magnocellular part	1	Contra
Male	4	−0.7	RSD	Retrosplenial dysgranular cortex	2	Contra
		−1.34	S1BF	Primary somatosensory cortex, barrel	1	Ipsi
		0.38	S1BF/S1ULp	Primary somatosensory cortex, barrel/primary somatosensory cortex, upper lip	1	Ipsi
		−0.7	S1HL	Primary somatosensory cortex, hindleg	1	Contra
		−6.97	Gi	Gigantocellular vestibular nucleus	1	Ipsi
Male	5	−5.23	PnC	Pontine reticular nucleus, caudal part	2	Contra
		−6.64	Gi	Gigantocellular vestibular nucleus	1	Contra
Male	6	−4.16	mRt	Mesencephalic reticular formation	1	Contra
		−6.0	LVe	Lateral vestibular nucleus	1	Contra
		−7.78	MdV	Medullary reticular nucleus, ventral part	1	Contra
Male	7	−5.20	PnC	Pontine reticular nucleus, caudal part	1	Contra
		−5.88	LPGi	Lateral paragigantocellular nucleus	1	

### The spinal *Glra3*-Cre populations receive monosynaptic information from multiple subgroups of primary afferents

**Figure 13.**
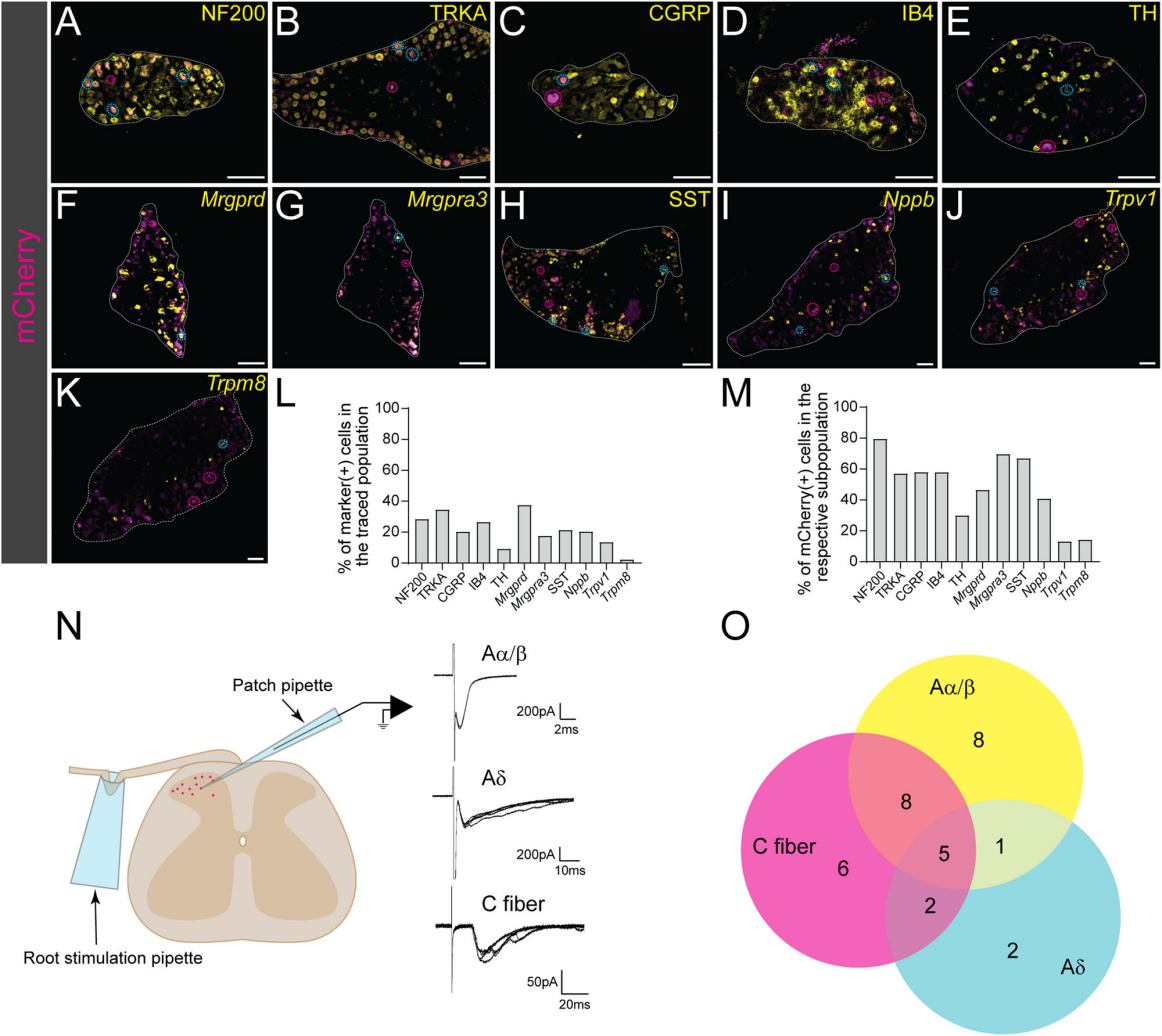
The spinal *Glra3*-Cre populations receive monosynaptic information from multiple subgroups of primary afferents. ***A–K***, Coexpression of markers (yellow) in lumbar DRG mCherry(+) traced cells (magenta). The cyan dotted circles indicate mCherry(+)marker(+) and the magenta dotted circles show examples of mCherry(+)marker(−) cells. ***A***, NF200: 2 females, 2 males. ***B***, TRKA: 2 females, 2 males. ***C***, CGRP: 1 female, 2 males. ***D***, IB4: 3 females, 1 male. ***E***, TH: 4 females, 2 males. ***F***, *Mrgprd*: 1 female, 2 males. ***G***, *Mrgpra3*: 1 female, 2 males. ***H***, SST: 2 females, 1 male. ***I***, *Nppb*: 1 female, 2 males. ***J***, *Trpv1*: 1 female, 2 males. ***K***, *Trpm8*: 1 female, 2 males. Scale bar, 100 µm. ***L***, Bar plot of the proportion of marker coexpression in mCherry(+) DRG cells. ***M***, Bar plot of the occurrence of mCherry(+) in marker-expressing DRG cells. The results are shown as total percentages of overlap. ***N***, Schematic illustration of a root stimulation combined patch-clamp recording, where red dots indicate *Glra3*-Cre(+) neurons. The traces in the middle are representative patch-clamp recordings of monosynaptic inputs from different afferent fibers. ***O***, Venn diagram illustrating the distribution of monosynaptic inputs from the different afferent fiber subtypes. The overlapping areas denote neurons that received monosynaptic inputs from multiple afferent fiber subtypes. For separate channels, see Figure 14.

**Figure 14.**
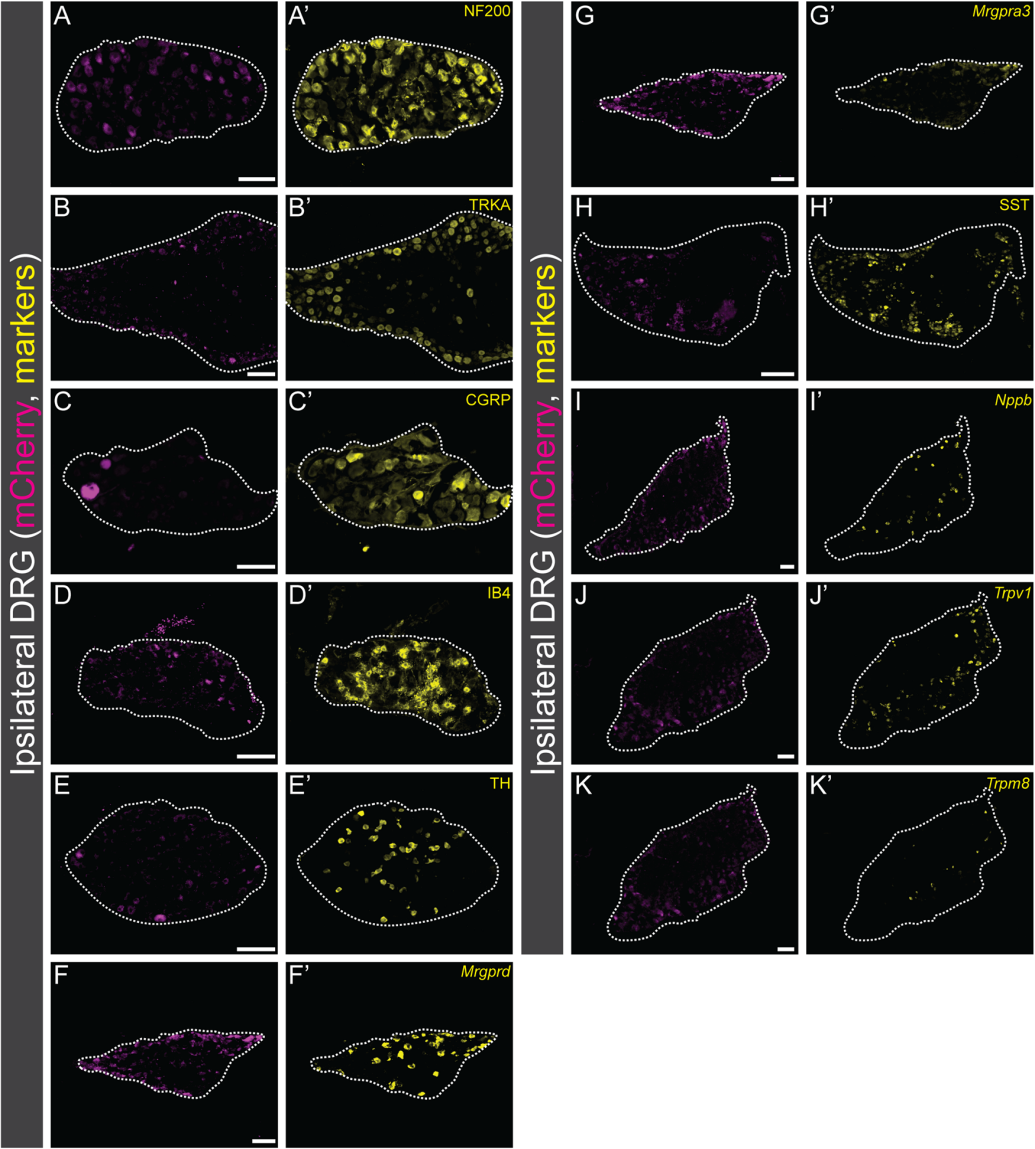
Spinal *Glra3*-Cre traced mCherry(+) colocalization with the marker genes and tested proteins, relating to Figure 13. Separate channel view of the different DRG markers from Figure 9. ***A–K***, The lumbar *Glra3*-Cre traced mCherry(+) cells in the DRG (magenta). ***A’–K’***, Marker genes and proteins. Scale bar, 100 µm.

Mono- and presynaptic traced mCherry(+) cells were detected in lumbar DRG of *Glra3*-Cre(+) mice, indicating that these spinal populations receive peripheral monosynaptic input. The traced cells were mainly found ipsilateral, but a few mCherry(+) cells were also detected in contralateral lumbar DRG in two out of six mice (*n* cells = 20). In *Glra3*-Cre(+) mice, traced cells were found in one ipsilateral thoracic DRG in two separate mice (*n* cells = 47). In one of these mice, and in a third mouse, mCherry(+) cells (*n* cells = 20) were found in one contralateral thoracic DRG. As mentioned above, mCherry(+) cells were observed in the contralateral DRG in two *Glra3*-Cre(−) mice, implying that the contralateral mCherry(+) cells found in the *Glra3*-Cre(+) mice may be false positives. To identify the *Glra3*-Cre(+) contacting primary afferents, we examined the overlap with the markers NF200, TRKA, CGRP, IB4, TH, *Mrgprd*, *Mrgpra3*, SST, *Nppb*, *Trpv1*, and *Trpm8* (Averill et al., 1995; Patapoutian et al., 2003; Li et al., 2011; Usoskin et al., 2015; Albisetti et al., 2017; Kupari and Ernfors, 2023; Fig. 13*A–N*, for separate channels, see Fig. 14). Of the ipsilateral lumbar DRG mCherry(+) cells, 28.4% (591/2,079) belonged to the neurofilament heavy myelinated NF200(+) group (Fig. 13*A*,*L*), which is present in Aδ- and Aα/β-fibers (Basbaum et al., 2009; Meltzer et al., 2021). CGRP is a pro-pruritic and noxious neuropeptide (McCoy et al., 2012; Rogoz et al., 2014), which is highly coexpressed with noxious receptor TRKA (Woolf et al., 1994; Averill et al., 1995; McCoy et al., 2012; Barker et al., 2020), and both genes have little overlap with IB4-binding fibers (Averill et al., 1995; McCoy et al., 2012; Usoskin et al., 2015). In total, 34.9% (687/1,988) of mCherry(+) cells overlapped with the TRKA(+) population (Fig. 14*B*,*L*) and 20.3% (213/1,049) with CGRP(+) (Fig. 13*C*,*L*). Furthermore, 26.6% (391/1,472) of the mCherry(+) cells overlapped with small unmyelinated nonpeptidergic neuronal binding marker IB4 (Fig. 14*D*,*L*), and 9.2% (179/1,951) with TH (Fig. 13*E*,*L*), which is expressed in low-threshold mechanosensory C-fibers (Li et al., 2011). In contrast, 79.5% (591/743) of the NF200(+), 57.1% (687/1,203) of TRKA(+), 58.0% (213/367) of CGRP(+), 57.9% (391/675) of IB4-binding, and 29.9% (179/598) of TH(+) neurons were mCherry(+) (Fig. 13*M*). Not all primary sensory afferents are equally susceptible to retrograde tracing by rabies virus (Albisetti et al., 2017); however, the overlap of the traced mCherry(+) neurons with all markers indicates that the spinal *Glra3*-Cre populations receive monosynaptic peripheral information from several fiber subtypes.

To obtain a more detailed view of the peripheral input to lumbar *Glra3*-Cre(+) neurons, we targeted markers for receptor and neurotransmitter DRG subtypes (Kupari and Ernfors, 2023; Fig. 13*F–M*). Three pruriceptive molecular clusters have previously been identified, namely, NP1, NP2, and NP3. *Mrgprd* is expressed in the NP1 cluster, *Mrgpra3* in the NP2, and SST/*Nppb* in the NP3 cluster (Usoskin et al., 2015), where *Sst* has little colocalization with IB4 (Usoskin et al., 2015; Stantcheva et al., 2016). *Mrgprd* was detected in 37.5% (147/392) (Fig. 13*F*,*L*), *Mrgpra3* in 17.6% (99/392; Fig. 14*G*,*L*), SST in 21.4% (265/1,239; Fig. 13*H*,*L*), and *Nppb* in 20.3% (72/354; Fig. 13*I*,*L*) of the traced mCherry(+) cells. Strikingly, mCherry was detected in a large portion of the pruriceptive subclusters: 46.5% (147/316) of *Mrgprd*(+), 69.7% (69/99) of *Mrgpra3*(+), 66.9% (265/394) of SST(+), and 40.9% (72/176) of *Nppb*(+) neurons (Fig. 14*M*), supporting the behavioral finding of the *Glra3*-Cre populations facilitating itch-related transmission. Lastly, the expressions of the temperature-sensitive channels *Trpv1* and *Trpm8* were investigated. *Trpv1* is activated by capsaicin and noxious temperatures (≥42°C), while *Trpm8* is activated by menthol and cooling temperatures (<26–28°C; Patapoutian et al., 2003). Herein, 13.5% (53/392) and 2.3% (9/392) of the *mCherry*(+) cells expressed *Trpv1* or *Trpm8*, respectively (Fig. 13*J–L*). In contrast, 13.2% (53/403) of the *Trpv1*(+) and 14.3% (9/63) of the *Trpm8*(+) populations expressed *mCherry* (Fig. 13*M*). The low overlap of *Trpv1* in traced neurons is consistent with both the low coexpression of *Glra3* in Hargreaves-induced *fos*(+) cells and the lack of phenotype of DREADD-mediated inactivation of *Glra3*-Cre(+) neurons in the same test.

To further investigate and validate that *Glra3*-Cre(+) neurons receive peripheral monosynaptic input, patch-clamp recordings were conducted on *Glra3*-Cre(+) [reporter tdTomato(+) and viral mCherry(+)] neurons in combination with dorsal root stimulation (Fig. 13*N*). The data revealed that the *Glra3*-Cre populations receive monosynaptic inputs from all afferent fiber subtypes. Half of the recorded neurons (16/32) received monosynaptic inputs from at least two afferent fibers, among which almost one-third (5/16) formed monosynaptic connections with all three fiber subtypes. Furthermore, the majority of synaptic inputs was delivered via Aα/β fibers (41%) or C-fibers (40%), while only 19% was transmitted by Aδ fibers (Fig. 13*O*). Collectively, these results confirmed that *Glra3*-Cre(+) neurons receive monosynaptic information from multiple afferent fiber subtypes, including myelinated and itch-associated neurons, suggesting that the *Glra3*-Cre populations form complex monosynaptic connections with primary afferents.

## Discussion

Herein, we report that the *Glra3*-Cre line labels excitatory and inhibitory primarily dorsal neuronal populations in the spinal cord that express *Glra3*. These populations respond to glycine and are heterogeneous in terms of AP firing patterns and homogenous in intrinsic membrane properties. Behavioral and expressional analyses revealed that spinal *Glra3*-Cre populations have a pro-pruritic role in compound 48/80- and chloroquine-evoked itch and no role in the mechanical or thermal responses that have been tested in this study. Analyses using monosynaptic retrograde tracing and dorsal root stimulations demonstrated that lumbar *Glra3*-Cre populations receive monosynaptic excitatory and inhibitory input from neurons within the lumbar division, several brain areas related to sensory and motor functions, and afferents belonging to the NF200(+), TRKA(+), IB4-binding, and TH(+) subpopulations. Furthermore, CGRP(+) and pruritic markers *Mrgprd*(+), *Mrgpra3*(+), SST(+), and *Nppb*(+) afferent populations were found to synapse on spinal *Glra3*-Cre(+) neurons. The multitude of sensory modality input to this population was confirmed with dorsal root stimulations. Taken together, the data show that the spinal *Glra3*-Cre populations communicate compound 48/80 and chloroquine-evoked itch.

The glycinergic system is a fast response inhibitory system important for modulating motor and sensory reflex activity, muscle tone, and respiratory rhythms (Manzke et al., 2010; Cioffi, 2018). The glycinergic system serves a protective role in pain and itch, where activation of glycinergic neurons leads to attenuated pain and itch responses, and ablation causes nociceptive and pruriceptive hypersensitivity (Foster et al., 2015). Blocking spinal glycine receptors decreases the nociceptive counterstimulation effect on persistent itch-mediated spontaneous activity in the spinal cord (Akiyama et al., 2011), implementing the importance of the glycinergic system in sensory regulation. Our chemogenetic activation experiments indicate that the adult spinal *Glra3*-Cre populations have an acute sensory role as its activation evoked spontaneous sensory behaviors, such as licking/biting, stomping, and guarding of the affected dermatome, whereas silencing decreased compound 48/80- and chloroquine-induced itch, indicative of a pro-pruritic role.

In the retrograde rabies tracing experiment, we investigated overlap of traced neurons with primary afferent subtype markers to deduce the sensory modality input to the spinal *Glra3*-Cre populations. Almost half of the NP1-*Mrgprd*(+) (β-alanine receptor; Liu et al., 2012) and NP3-*Nppb*(+)/*Sst*(+) (Usoskin et al., 2015; Stantcheva et al., 2016) primary afferents synapse on *Glra3*-Cre(+) neurons. Activation of SST(+) primary afferents evokes pruritofensive behaviors and deletion of *Sst* attenuates itch evoked by pruritogens, such as compound 48/80 and chloroquine (Huang et al., 2018). Furthermore, SST(+) primary afferent ablation decreases histamine, chloroquine, IL-31- and serotonin-evoked scratching (Stantcheva et al., 2016). Herein, we found that almost 70% of SST(+) primary afferents and chloroquine receptor *Mrgpra3*(+) primary afferents, found in the NP2 cluster (Liu et al., 2009; Usoskin et al., 2015), synapse on *Glra3*-Cre(+) neurons. TRKA is the receptor of NGFβ, a neurotrophic protein important for hyperalgesia (Woolf et al., 1994; Barker et al., 2020) and CGRP is a neuropeptide with pro-pruritic and noxious functions (McCoy et al., 2012; Rogoz et al., 2014). *Ntrk1* (gene encoding TRKA) and *Calca* (gene encoding CGRP) are highly coexpressed in nonpeptidergic pruriceptive NP2 neurons and in nociceptive peptidergic PEP1–2 neurons. Our retrograde monosynaptic tracing showed that *Glra3*-Cre(+) neurons receive monosynaptic input from TRKA(+) and CGRP(+) primary afferents. The dorsal root stimulation further confirmed that these populations receive monosynaptic input from C-fibers, collectively implying that spinal *Glra3*-Cre(+) neurons are central for communicating itch.

Transcriptional validation of the behavioral involvement of spinal *Glra3*-Cre(+) neurons in different sensory modalities confirmed that *Glra3* is largely expressed in compound 48/80-evoked *fos*(+) neurons compared with saline-induced *fos*(+) cells. Compared with the contralateral side, chloroquine-activated *fos*(+) cells expressed *Glra3* but this effect could not be separated from the influence of the injection itself. However, the chloroquine-activated cells constitute a smaller population than the saline-activated group (*p* < 0.0001), which may explain this result. Previous studies have found that itch-inducing compounds activate cells in the superficial dorsal horn (Yao et al., 1992; Doi-Saika et al., 1997; Jinks and Carstens, 2000; Nojima et al., 2003; Nakano et al., 2008; Han et al., 2012; Akiyama et al., 2013; Gatto et al., 2021), which is similar to our findings.

Consistent with the absence of thermal response alterations following *Glra3* deletion/mutation observed by the cited studies (Harvey et al., 2009; Werynska et al., 2021), chemogenetic silencing of *Glra3*-Cre neurons did not alter the withdrawal response in the Hargreaves test. Subsequent histological analysis showed that Hargreaves-activated *fos*(+) cells did not overlap with *Glra3* compared with the contralateral side in naive mice. Moreover, silencing did not affect the response in the acetone drop test, further dismissing involvement of the spinal *Glra3*-Cre(+) neurons in acute thermal transmission. The retrograde rabies tracing revealed that the *Glra3*-Cre populations receive sparse monosynaptic input from *Trpm8*(+) primary afferents, while 13.5% of the traced DRG neurons overlapped with *Trpv1*. TRPV1(+) primary afferents are key mediators in itch transmission (Mishra et al., 2011; Rogoz et al., 2014) and TRPV1-deficient mice show reduced responses to histamine (Imamachi et al., 2009). The *TrpV1(+)* primary afferent input to the lumbar *Glra3*-Cre populations may therefore be related to itch rather than thermal sensation. Silencing of GLYT2 neurons do however regulate both mechanical and thermal transmission (Foster et al., 2015) and activation of GLYT2 neurons has an antihyperalgesic effect on neuropathic-induced mechanical allodynia (Foster et al., 2015). Meanwhile, deletion/mutation of *Glra3* does not affect the withdrawal response to mechanical and thermal stimulation following nerve injury (Harvey et al., 2009; Werynska et al., 2021). Chemogenetic silencing of the *Glra3*-Cre populations did not affect the acute mechanical sensitivity in the Randall–Selitto test and scratch- or pinch-activated *fos*(+) cells did not express *Glra3* in higher occurrence compared with the contralateral side in naive mice. Conclusively, our analyses indicate that the *Glra3*-Cre(+) neurons may not be the postsynaptic target of the GLYT2 population in regulation of noxious mechanical and thermal transmission. However, since GLRA3 has been connected to inflammatory-induced hypersensitivity (Harvey et al., 2009; Werynska et al., 2021), future investigations targeting the role of *Glra3*-Cre(+) neurons in inflammatory, neuropathic, thermal, and mechanical allodynia are of interest.

Besides input from itch-related primary afferents, the monosynaptic tracing experiments and dorsal root stimulations revealed that the *Glra3*-Cre populations receive input from Aα∕β fibers as partial overlap with NF200(+). As NF200 can be detected in Aβ low-threshold mechanoreceptors (LTMRs), Aβ high-threshold mechanoreceptors (HTMRs), and Aδ-fibers (Djouhri and Lawson, 2004; Nagi et al., 2019; Meltzer et al., 2021), input from these neuronal subpopulations cannot be excluded. Furthermore, the overlap of mCherry(+) cells with TH(+) neurons, which convey low-threshold mechanical information and are possibly associated with pleasant touch (Li et al., 2011), proposes that the lumbar *Glra3*-Cre populations receive several categories of sensory input. In addition, traced cells were found in the ventral horn (laminae VII-IV), indicating that the *Glra3*-Cre populations may receive input from spinal motor-related neurons. Additionally, starter, lineage, and virally labeled *Glra3*-Cre(+) cells were observed in the ventral horn. In line with these observations, we recently showed that *Glra3* is detected in the dorsal and ventral horns of the lumbar division (Ceder et al., 2023). Thus, it remains unclear whether sensory-mediating *Glra3*-Cre(+) neurons receive motor input or if the ventrally located *Glra3*-Cre population have motor functions.

Traced cells were also detected in the brain, suggesting that the lumbar *Glra3*-Cre(+) neurons receive distant descending input. These brain areas included the contralateral motor cortices, ipsilateral primary somatosensory cortex, barrel area, contralateral p1 reticular formation, magnocellular and parvicellular parts of the red nucleus (RMC and RPC), ipsilateral oral and caudal part of the pontine reticular nucleus, and bilateral gigantocellular vestibular nucleus. Previous unilateral retrograde tracing from the cervical 1 and 2 segments in mouse shows a similar tracing pattern as observed in our tracing experiment (Liang et al., 2011). The RMC and reticular formations are related to analgesic functions (Prado et al., 1984; Martins and Tavares, 2017; Basile et al., 2021) and the RMC, RPC, and pontine reticular nucleus to motor functions (Morales et al., 1987; Kennedy, 1990; Basile et al., 2021). Moreover, a study in mice linked monosynaptic signaling from the motor and sensory cortices to distinct spinal dorsal and ventral interneuron populations and further to different motoric functions. Here, scant monosynaptic inputs from the motor cortex to dorsal horn neurons and from the sensory cortex to ventral neurons were observed (Ueno et al., 2018), indicating that the ventrally located *Glra3*-Cre(+) neurons probably receive monosynaptic input from the motor cortex. Collectively, we showed that the spinal *Glra3*-Cre populations receive monosynaptic descending input from brain areas involved in sensory and/or motor functions.

### Conclusions

Spinal GLYT2 neurons regulate itch (Foster et al., 2015), suggesting that the glycinergic system has potential as a drug target for itch. Nonetheless, thus far, the pruriceptive roles of the glycine receptor subunits have not been evaluated. Here, we successfully linked the *Glra3*-Cre populations to a pro-pruriceptive role in itch, indicating that GLRA3 may be a potential novel target for itch treatment. The spontaneous guarding behaviors observed from activating the *Glra3*-Cre populations are indicative of a role in sensory hypersensitivity (Wang and Wang, 2003; Mogil and Crager, 2004; Casarrubea et al., 2019) and raises questions regarding the hypersensitivity involvement of these populations for future investigations.

#### Methodological considerations

From the monosynaptic retrograde viral tracing, the lumbar *Glra3*-Cre populations were found to receive both inhibitory PAX2(+) and presumably excitatory, PAX2(−) input, from the lumbar segments, where the majority of the traced mCherry(+) cells were PAX2(−). However, the NEUN overlap analysis revealed that 44% of starter cells, 79% of traced mCherry(+) cells, and 89% of virally marked *Glra3*-Cre.mCherry were NEUN(+), which can be compared with the 98% NEUN(+) overlap in the *Glra3*-Cre;*tdTomato* cells. The decrease in overlap may indicate that the viral injections affect expressional patterns in the infected cells, and therefore, the PAX2(+) overlap in the starter and traced cells may be underestimated.
